# Metabolic syndrome and risk of sepsis and sepsis-related mortality: evidence from two large prospective cohort studies

**DOI:** 10.1016/j.mmr.2026.100031

**Published:** 2026-04-27

**Authors:** Shao-Yu Liu, Shao-Kang Xu, Jing-Li Gao, Xiao-Ke Kong, Jian Shi, Ya Miao, Yi-Ting Tang, Bin Zhao, Fang Fang, Ai-Tian Wang, Shou-Ling Wu, Jia-Qi Huang, Ben Lu

**Affiliations:** a Department of Critical Care Medicine and Hematology, the Second Xiangya Hospital, Central South University, Changsha 410011, China; b Department of Critical Care Medicine and Hematology, the Third Xiangya Hospital, Central South University, Changsha 410011, China; c Department of Intensive Medicine, Kailuan General Hospital, Tangshan 063000, Hebei, China; d National Clinical Research Center for Endocrine and Metabolic Diseases, Metabolic Syndrome Research Center, Key Laboratory of Diabetes Immunology, Ministry of Education, and Department of Metabolism and Endocrinology, the Second Xiangya Hospital of Central South University, Changsha 410011, China; e CSU-Sinocare Research Center for Nutrition and Metabolic Health, Xiangya School of Public Health, Central South University; Furong Laboratory, Changsha 410011, China; f Hunan Provincial Clinical Research Center for Sepsis, the Third Xiangya Hospital, Central South University, Changsha 410011, China; g Department of Physiology, Xiangya School of Basic Medical Science, Central South University, Changsha 410011, China; h Key Laboratory of Sepsis Translational Medicine of Hunan, Central South University, Changsha 410011, China; i Institute of Environmental Medicine, Karolinska Institutet, Stockholm 171 77, Sweden; j Department of Cardiology, Kailuan General Hospital, Tangshan 063000, Hebei, China

**Keywords:** Metabolic syndrome (MetS), Sepsis, Inflammation, Mortality, UK Biobank, Kailuan Study

## Abstract

**Background:**

Metabolic syndrome (MetS) is characterized by chronic low-grade inflammation and immune dysregulation, which may increase susceptibility to sepsis. However, epidemiologic evidence remains limited. This study aimed to evaluate the association of MetS with the risk of sepsis and sepsis-related mortality.

**Methods:**

This study included 359,633 participants from the UK Biobank and 152,317 participants from the Kailuan Study. MetS was defined as the presence of ≥3 metabolic abnormalities. Multivariable Cox proportional hazards models were used to estimate hazard ratios (*HRs*) and 95% confidence intervals (CIs) for the associations of MetS with risk of sepsis and 28-day mortality following sepsis. Stratified analyses were conducted to assess potential effect modification. In the UK Biobank, we further evaluated the dose-response relationship between the number of MetS components and sepsis outcomes, explored potential mediation by inflammatory and immune biomarkers, and investigated the joint effect of MetS and lifestyle; in the Kailuan Study, we further investigated the impact of MetS evolution on sepsis risk. Sensitivity analyses were performed to evaluate the robustness of the results.

**Results:**

During a median follow-up of 13.7 years, 11,040 sepsis cases were identified in the UK Biobank, whereas 5672 cases were documented in the Kailuan Study during a median follow-up of 16.4 years. After multivariable adjustment, MetS was associated with higher risks of sepsis (*HR*=1.55, 95% CI 1.49–1.61) and 28-day mortality following sepsis (*HR*=1.51, 95% CI 1.37–1.65) in the UK Biobank; corresponding *HRs* were 1.32 (95% CI 1.25–1.40) and 1.49 (95% CI 1.32–1.69) in the Kailuan Study, respectively (all *P*<0.001). These associations were generally consistent across stratified analyses. Moreover, the risk of sepsis outcomes increased with the number of MetS components and was partly mediated by inflammation. Compared with individuals free of MetS, individuals with MetS and an unfavorable lifestyle had substantially higher risks of sepsis (*HR*=1.91, 95% CI 1.81–2.00) and 28-day mortality following sepsis (*HR*=1.84, 95% CI 1.64–2.07), whereas those with MetS but a favorable lifestyle showed only a modestly increased risk of sepsis and no excess risk of 28-day mortality (*HR*=1.18, 95% CI 1.09–1.28 and *HR*=1.05, 95% CI 0.88–1.27, respectively). In analyses of MetS evolution, using individuals with persistently normal metabolic status as the reference, those with a persistent MetS demonstrated the highest risks of sepsis (*HR*=1.46, 95% CI 1.32–1.61) and 28-day mortality following sepsis (*HR*=1.88, 95% CI 1.50–2.35), followed by individuals with progressive MetS (*HR*=1.17, 95% CI 1.05–1.31 and *HR*=1.36, 95% CI 1.04–1.77, respectively), whereas those who recovered from MetS did not show a significantly increased risk (*HR*=1.09, 95% CI 0.96–1.25 and *HR*=1.19, 95% CI 0.87–1.61, respectively). Sensitivity analyses confirmed the robustness of the findings.

**Conclusions:**

This study demonstrated that MetS was associated with an increased risk of sepsis and sepsis-related mortality. These associations were partially mediated through inflammatory responses. The findings highlight the importance of maintaining metabolic health as well as promoting healthy lifestyles as strategies to reduce its burden.

## 1 Background

Sepsis is a severe organ dysfunction resulting from dysregulated immune responses to infection, and it remains a predominant cause of mortality and disability globally [1]. The Global Burden of Disease Study estimates that approximately 48.9 million individuals develop sepsis worldwide, leading to 11 million fatalities (i.e., nearly 20% of all global deaths) [2]. This underscores the importance of preventative approaches to reduce the incidence of sepsis and its resultant mortality, emphasizing an unmet need to identify risk factors for the onset and progression of sepsis [3].

Metabolic syndrome (MetS) is characterized by a collection of interrelated cardiometabolic risk factors, including central obesity, high blood pressure, dyslipidemia, and impaired glucose metabolism [4], which collectively contribute to a chronic low-grade inflammatory state and immune dysregulation [5], [6]. Notably, the burden of MetS has increased steadily over the past three decades. Among US adults, the prevalence of MetS increased from approximately 25.3% in 1988–1994 to 34.2% in 2007–2012, and further to 38.5% in 2021–2023 [7], [8]. In addition, MetS has been widely recognized as a key risk factor for mortality and the development of chronic diseases, including cardiovascular disease, cancer, and dementia [9], [10], [11], [12]. Prior studies have demonstrated that individual metabolic traits, such as obesity, dysregulated glucose metabolism, and dyslipidemia, are associated with risk of severe infection [13], [14], [15]. However, these metabolic abnormalities often co-occur and collectively shape host defense. A single-component perspective may therefore underestimate the integrated immunometabolic vulnerability that arises when multiple perturbations co-exist. Thus, evaluating MetS as the exposure extends beyond individual metabolic traits to capture the clustering and cumulative burden of metabolic disturbances, which may jointly predispose an individual to sepsis through systemic immune-metabolic dysregulation. Several studies have also suggested an association between MetS and an increased risk of acute health outcomes, primarily among hospitalized or critically ill patients [16], [17], [18]. However, a potential link between MetS and sepsis has rarely been investigated, especially in the general population.

To address this knowledge gap, we examined the association of MetS with the risk of incident sepsis and sepsis-related mortality across two large prospective cohorts, the UK Biobank and the Kailuan Study. In the UK Biobank, we also evaluated the dose-response relationship between the number of MetS components and sepsis outcomes, potential mediating roles of inflammatory biomarkers, and the joint effect of MetS and lifestyle. In the Kailuan Study, we additionally investigated the impact of dynamic changes in MetS status on sepsis outcomes. This study aimed to comprehensively evaluate the associations of MetS with risk of sepsis and sepsis-related mortality.

## 2 Methods

### 2.1 Study population

In this study, we used data from two large prospective cohorts, the UK Biobank and the Kailuan Study, to assess the potential role of MetS on the risk of sepsis incidence and 28-day mortality following sepsis. The UK Biobank is a large-scale prospective cohort study that recruited over 500,000 participants aged 37 to 73 years between 2006 and 2010 across 22 assessment centers in England, Wales, and Scotland [19]. At recruitment, comprehensive health-related data were collected via touchscreen questionnaires, physical assessment, biological sample collection, and electronic health records. All participants provided written informed consent, and the study received approval from the North West Multi-Centre Research Ethics Committee (11/NW/03820). The Kailuan Study is an ongoing, community-based prospective cohort study conducted in Tangshan, China. Since 2006, it has enrolled more than 150,000 participants, primarily current and retired employees of the Kailuan community, who undergo standardized health examinations at recruitment and biennially thereafter. The study design has been described in detail elsewhere [20], [21]. All participants completed standardized questionnaires and underwent health examinations (including clinical and laboratory assessments) at 11 hospitals that provide healthcare services to the Kailuan community. The study protocol was approved by the Ethics Committee of Kailuan General Hospital (2006–05), and written informed consent was obtained from all participants.

In both the UK Biobank and the Kailuan Study, we excluded participants with a history of cancer or sepsis at baseline and those with missing information on the components of MetS. In the UK Biobank, we also excluded individuals who withdrew from the study or were pregnant at baseline. In the Kailuan Study, we also excluded participants with missing baseline demographic information. The final analytic cohorts consisted of 359,633 participants in the UK Biobank and 152,317 in the Kailuan Study (**Additional file 1:** Fig. S1).

For the analysis of MetS evolution, we identified a sub-cohort of 62,578 participants who completed both the 2006 and 2010 health examinations, including complete data on MetS components at these two occasions in the Kailuan Study (**Additional file 1:** Fig. S2).

### 2.2 Exposure assessment

MetS was defined based on the Harmonized Criteria established in 2009 by the International Diabetes Federation (IDF) and the American Heart Association/National Heart, Lung, and Blood Institute (AHA/NHLBI) (**Additional file 1:** Table S1) [22]. In the UK Biobank, we used different data sources, such as medication codes and serum biomarker concentrations, to determine the presence of MetS at baseline, according to the methodology developed from a previous study (**Additional file 1:** Table S2) [23]. In the Kailuan Study, MetS-related information was collected from laboratory measurements, self-reported questionnaire data on medication use, and physical examination (e.g., blood pressure and anthropometric measures) [24]. A diagnosis of MetS was given for participants with at least three of the following five MetS components: 1) elevated waist circumference (≥102 cm in men and ≥88 cm in women in the UK Biobank; ≥85 cm in men and ≥80 cm in women in the Kailuan Study); 2) hypertriglyceridemia (≥150 mg/dl or 1.7 mmol/L); 3) elevated blood pressure (≥130 mmHg systolic blood pressure and/or ≥85 mmHg diastolic blood pressure or antihypertensive medication use); 4) hyperglycemia [glycated hemoglobin A1c (HbA1c) ≥39 mmol/mol or treatment for high blood glucose in the UK Biobank; fasting plasma glucose >100 mg/dl or treatment for high blood glucose in the Kailuan Study]; and 5) reduced high-density lipoprotein (HDL) cholesterol levels (<40 mg/dl or 1.0 mmol/L in men and <50 mg/dl or 1.3 mmol/L in women or the use of lipid‐modifying medications). Given that circulating glucose levels were measured predominantly in non-fasting samples, we defined hyperglycemia using HbA1c as a surrogate marker in the UK Biobank, based on the American Diabetes Association-recommended threshold [25], [26].

In the UK Biobank, we examined the potential joint effect between MetS and lifestyle. We generated an unhealthy lifestyle score based on information collected at recruitment, including body mass index (BMI), smoking, alcohol consumption, diet quality, physical activity, and sleep duration, according to the previous study [27]. The participants were then categorized into three groups based on the tertiles of the score: favorable (score <2), intermediate (score =2), or unfavorable (score >2) lifestyle (additional details are provided in **Additional file 1: Methods**).

Finally, we assessed MetS evolution in the sub-cohort as described above in the Kailuan Study. Participants were classified into 4 groups according to their MetS status at these two time points. 1) sustained metabolic health: no MetS in either 2006 or 2010; 2) MetS progression: no MetS in 2006 but MetS in 2010; 3) MetS recovery: MetS in 2006 but not in 2010; and 4) persistent MetS: MetS in both 2006 and 2010 [24].

### 2.3 Assessment of sepsis outcomes

The primary outcomes were incident sepsis and 28-day mortality following sepsis (as a severe sepsis-related event). In the UK Biobank, diagnosis of sepsis was primarily ascertained through the hospital inpatient records and death registers, based on the 9th and the 10th revisions of the International Classification of Diseases (ICD-9 and -10) (**Additional file 1:** Table S3) [28]. In addition to the primary outcomes, we also studied mortality at 7-day, 60-day, 90-day, 180-day, and 1-year following sepsis as secondary outcomes. Follow-up time was calculated from the date of recruitment until the occurrence of the outcome of interest (i.e., sepsis or 28-day mortality following sepsis), loss to follow-up, death, or the end of follow-up (October 31, 2022), whichever came first.

In the Kailuan Study, incident sepsis was identified using complementary approaches. For participants with detailed clinical records, sepsis was ascertained according to the Third International Consensus Definitions for Sepsis and Septic Shock (Sepsis-3), namely, suspected or documented infection with concurrent organ dysfunction defined by an increase in Sequential Organ Failure Assessment (SOFA) score of ≥2 points [1], [29]. For participants without clinical record data, sepsis was identified through hospital discharge diagnoses based on ICD codes, including both explicit sepsis codes and implicit sepsis definitions (i.e., infection codes combined with organ dysfunction codes) (**Additional file 1:** Tables S4, S5**;** additional details are provided in **Additional file 1: Methods**) [30], [31]. Information was obtained from the 11 hospitals providing healthcare services to the Kailuan community, as well as the municipal health insurance and death registries. Follow-up time was calculated from the date of recruitment (i.e., the first health examination) until the occurrence of the outcome of interest, loss to follow-up, death, or December 31, 2023, whichever came first. For the analysis of MetS evolution in the sub-cohort, follow-up time was calculated from the date of the 2010 examination to the occurrence of the outcome of interest (i.e., sepsis or 28-day mortality following sepsis), loss to follow-up, death, or December 31, 2023, whichever came first.

### 2.4 Covariates

In the UK Biobank, information on sociodemographic and lifestyle factors was collected through touchscreen questionnaires, including age (continuous variable), sex (men or women), assessment center (categorical variable), ethnicity (White or others), Townsend deprivation index (TDI; continuous variable), smoking status (never, former, or current smoker), alcohol consumption (≤14 or >14 units/week), healthy diet (<5 or ≥5 points), educational level (high, intermediate, or low qualifications), regular physical activity (yes or no), sleep duration (short: <7 h/d; normal: 7 to 8 h/d; or long: >8 h/d). Alcohol consumption was categorized in accordance with the guidelines of the National Health Service [32]. A healthy diet score was developed to evaluate the level of adherence to an overall healthy dietary pattern, including 10 dietary factors, according to established nutritional recommendations (**Additional file 1:** Table S6) [33]. Physical activity was quantified using the total metabolic equivalent score, derived from the International Physical Activity Questionnaire short form. Regular physical activity was defined as engaging in at least 150 min of moderate-intensity activity per week, 75 min of vigorous-intensity activity per week, or an equivalent combination of both. Additionally, individuals were considered regularly physically active if they participated in moderate-intensity activity at least 5 d per week or in vigorous-intensity activity at least 1 d per week [34]. We also included the use of medications, including non-steroidal anti-inflammatory drugs (NSAIDs, i.e., aspirin and non-aspirin NSAIDs), and supplements, including vitamins and mineral and other dietary supplements, as covariates. Vitamin supplementation was defined by regular intake of vitamin A, B, C, D, or E, folic acid, or a multivitamin. Mineral and other dietary supplementation was defined by regular intake of glucosamine, fish oil, calcium, zinc, iron, or selenium.

In the Kailuan Study, information on sociodemographic and lifestyle factors was collected by trained staff through face-to-face interviews using a standardized questionnaire. We included age (continuous variable), sex (men or women), educational level (high school or above or less than high school), assessment center (categorical variable), smoking status (current or not), alcohol consumption (current or not), physical activity (low, moderate, or high), and sleep duration (<7 h/d, 7 to 8 h/d, or >8 h/d) as covariates.

For covariates with missing data (<7% in the UK Biobank and <9% in the Kailuan Study), imputation was conducted using the mode for categorical variables and the median for continuous variables based on the final analytical cohort (**Additional file 1:** Tables S7, S8).

### 2.5 Statistical analysis

Given the substantial differences in population composition and healthcare context between the UK Biobank and the Kailuan Study, the analyses were conducted separately within each cohort. We examined incidence rates per 1000 person-years across MetS strata in both cohorts. Continuous variables are presented as mean±standard deviation (SD), and categorical variables are presented as *n* (%). Cox proportional hazards regression models were used to estimate hazard ratios (*HRs*) and 95% confidence intervals (CIs) for the associations between MetS and risk of incident sepsis and 28-day mortality following sepsis. In the UK Biobank, Model 1 was adjusted for age and sex. Model 2 was additionally adjusted for lifestyle and sociodemographic factors, including ethnicity, TDI, smoking status, alcohol consumption, educational level, healthy diet, regular physical activity, and sleep duration. Model 3 further accounted for the use of aspirin, non-aspirin NSAIDs, vitamins, and mineral and other dietary supplements. In the Kailuan Study, Model 1 was adjusted for age and sex. Model 2 was additionally adjusted for educational level, assessment center, smoking status, alcohol consumption, physical activity, and sleep duration.

We performed stratified analyses to assess potential effect modification of the studied associations. In the UK Biobank, subgroups were defined according to age (<60 or ≥60 years), sex (men or women), educational level (college/university degree or not), TDI (below or above median), current smoking (no or yes), alcohol drinking (≤14 or >14 units/week), healthy diet (no or yes), regular physical activity (no or yes), sleep duration (normal or abnormal), and years of follow-up (<5, 5–10, or ≥10 years). In the Kailuan Study, stratified analyses were performed by age (<60 or ≥60 years), sex (men or women), educational level (high school or above or less than high school), current smoking (no or yes), current alcohol consumption (no or yes), physical activity (low, moderate, or high), and sleep duration (normal or abnormal). We calculated the statistical significance of the interactions between MetS and these variables via likelihood ratio tests, comparing Cox proportional hazards regression models with and without the respective interaction term. The proportional hazards assumption was tested using Schoenfeld residuals, and no major violation was found.

In the UK Biobank, to assess the contribution of individual MetS components, we estimated *HRs* for each of the five components, and to examine the dose-response relationship, we analyzed the risk of sepsis outcomes in relation to the number of MetS components (as a proxy for the severity of MetS). Accumulating evidence has suggested that MetS and its components promote chronic low-grade inflammation and are associated with elevated levels of pro-inflammatory factors [35], [36]. MetS has also been linked to suppression of the insulin-like growth factor-1 (IGF-1) axis, which exerts important immunomodulatory and anti-inflammatory effects [37]. Dysregulated host immune function and uncontrolled inflammatory response to infection are central pathogenic mechanisms underlying sepsis development [38]. Therefore, several biomarkers were selected and tested as potential mediators for the association between MetS and sepsis risk, including C-reactive protein (CRP) level, white blood cell count, neutrophil count, monocyte count, platelet-to-lymphocyte ratio (PLR), systemic immune-inflammation index (SII), lymphocyte count, and IGF-1 level. The %MEDIATE SAS macro was used to compute the mediation proportion and its 95% CI for each factor. To examine the joint effect between MetS and lifestyle, we compared the risk of sepsis and 28-day mortality following sepsis among individuals with MetS and unfavorable, intermediate, or favorable lifestyles to those without MetS (i.e., regardless of lifestyle). Finally, we evaluated the associations between MetS and risk of mortality at 7-day, 60-day, 90-day, 180-day, or 1-year post-sepsis. In the sub-cohort of the Kailuan Study, we examined the impact of persistent MetS on sepsis by assessing the associations between MetS evolution and sepsis outcomes.

Several sensitivity analyses were performed to evaluate the robustness of the findings (details are provided in **Additional file 1: Methods**). To assess whether specific MetS phenotypes confer disproportionately elevated risk, we evaluated the associations between each of the 16 possible combinations of MetS components and subsequent risk of sepsis outcomes. A directed acyclic graph was constructed to describe the hypothesized causal relationships among exposure, outcome, covariates, and potential intermediate processes (**Additional file 1:** Fig. S3). All analyses were performed using SAS version 9.4 software (SAS Institute, Cary, North Carolina, USA) and R version 4.4.1 (R Foundation for Statistical Computing, Vienna, Austria). All statistical analyses were two-sided, using a type I error rate of 0.05. Based on previous studies and a priori estimate, a two-sided *P*-value threshold of 0.005 was set to determine statistical significance and account for multiple comparisons [39].

## 3 Results

### 3.1 Baseline characteristics

A total of 511,950 participants (359,633 from the UK Biobank and 152,317 from the Kailuan Study) were included in the present analysis. Baseline characteristics of the study participants are presented in Table 1, Table 2. Overall, 32.2% of the participants met the diagnostic criteria for MetS [32.6% (117,062/359,633) in the UK Biobank and 31.4% (47,763/152,317) in the Kailuan Study]. The mean age at baseline was (56.2±8.1) years in the UK Biobank and (48.4±14.3) years in the Kailuan Study. In both cohorts, participants with MetS were generally older, more likely to be men, had a lower educational level, and more frequently reported short or long sleep duration compared with other participants. In the UK Biobank, individuals with MetS also had a higher TDI score, were more often current smokers, less likely to adhere to a healthy diet, and engaged less in physical activity. In the Kailuan Study, the prevalences of smoking, alcohol consumption, and physical activity were generally similar between participants with and without MetS.

**Table 1:** Baseline characteristics of the study participants according to the status of metabolic syndrome (MetS) in the UK Biobank.

**Characteristics**	**Overall**	**No MetS**	**MetS**	***P*****-value**
No. of participants	359,633	242,571	117,062	
Age [year, mean±SD]	56.2±8.1	55.2±8.1	58.4±7.6	<0.001
Male [*n* (%)]	171,197 (47.6)	108,642 (44.8)	62,555 (53.4)	<0.001
White [*n* (%)]	340,817 (94.8)	231,286 (95.4)	109,531 (93.6)	<0.001
Education [*n* (%)]^a^				<0.001
High qualifications	117,004 (32.5)	88,156 (36.3)	28,848 (24.6)	
Intermediate qualifications	182,566 (50.8)	121,836 (50.2)	60,730 (51.9)	
Low qualifications	60,063 (16.7)	32,579 (13.4)	27,484 (23.5)	
Townsend deprivation index [mean±SD]	–1.3±3.1	–1.5±3.0	–1.1±3.2	<0.001
Smoking status [*n* (%)]				<0.001
Never smoker	199,183 (55.4)	141,324 (58.3)	57,859 (49.4)	
Former smoker	122,506 (34.1)	77,291 (31.9)	45,215 (38.6)	
Current smoker	37,944 (10.6)	23,956 (9.9)	13,988 (11.9)	
Alcohol consumption >14 units/week [*n* (%)]	118,957 (33.1)	82,579 (34.0)	36,378 (31.1)	<0.001
Healthy diet [*n* (%)]^b^	53,541 (14.9)	37,418 (15.4)	16,123 (13.8)	<0.001
Drug or supplement use [*n* (%)]				
Aspirin	49,559 (13.8)	20,614 (8.5)	28,945 (24.7)	<0.001
Non-aspirin non-steroidal anti-inflammatory drugs	53,206 (14.8)	36,938 (15.2)	16,268 (13.9)	<0.001
Vitamins	112,185 (31.2)	78,146 (32.2)	34,039 (29.1)	<0.001
Mineral and other dietary supplements	152,360 (42.4)	103,758 (42.8)	48,602 (41.5)	<0.001
Regular physical activity [*n* (%)]	282,950 (78.7)	196,402 (81.0)	86,548 (73.9)	<0.001
Sleep duration [*n* (%)]				<0.001
Short (<7 h/d)	88,154 (24.5)	57,101 (23.5)	31,053 (26.5)	
Normal (7–8 h/d)	244,956 (68.1)	170,241 (70.2)	74,715 (63.8)	
Long (>8 h/d)	26,523 (7.4)	15,229 (6.3)	11,294 (9.6)	
Component of MetS [*n* (%)]				
Elevated waist circumference	119,697 (33.3)	34,786 (14.3)	84,911 (72.5)	<0.001
Hypertriglyceridemia	144,257 (40.1)	54,844 (22.6)	89,413 (76.4)	<0.001
Elevated blood pressure	253,593 (70.5)	144,396 (59.5)	109,197 (93.3)	<0.001
Hyperglycemia	65,838 (18.3)	13,980 (5.8)	51,858 (44.3)	<0.001
Reduced HDL‐cholesterol	113,975 (31.7)	32,234 (13.3)	81,741 (69.8)	<0.001

a High qualifications (college or university degree), intermediate qualifications (advanced/advanced subsidiary levels or equivalent or ordinary levels/general certificate of secondary education or equivalent or certificate of secondary education or equivalent or national vocational qualification or higher national diploma or higher national certificate or equivalent or other professional qualifications), and low qualifications (none of the above).

b Healthy diet: healthy diet score ≥5. HDL. High-density lipoprotein; SD. Standard deviation

**Table 2:** Baseline characteristics of the study participants according to the status of metabolic syndrome (MetS) in the Kailuan Study.

**Characteristics**	**Overall**	**No MetS**	**MetS**	***P*****-value**
No. of participants	152,317	104,554	47,763	
Age [year, mean±SD]	48.4±14.3	46.6±14.6	52.3±12.9	<0.001
Male [*n* (%)]	125,057 (82.1)	84,691 (81.0)	40,366 (84.5)	<0.001
High school or above [*n* (%)]	35,371 (23.2)	27,052 (25.9)	8319 (17.4)	<0.001
Current smoker [*n* (%)]	52,255 (34.3)	35,936 (34.4)	16,319 (34.2)	0.440
Current drinker [*n* (%)]	56,849 (37.3)	38,942 (37.2)	17,907 (37.5)	0.361
Physical activity [*n* (%)]				<0.001
Low	23,146 (15.2)	16,209 (15.5)	6937 (14.5)	
Moderate	107,478 (70.6)	74,136 (70.9)	33,342 (69.8)	
High	21,693 (14.2)	14,209 (13.6)	7484 (15.7)	
Sleep duration [*n* (%)]				<0.001
Short (<7 h/d)	39,773 (26.1)	26,776 (25.6)	12,997 (27.2)	
Normal (7–8 h/d)	109,898 (72.2)	75,924 (72.6)	33,974 (71.1)	
Long (>8 h/d)	2646 (1.7)	1854 (1.8)	792 (1.7)	
Component of MetS [*n* (%)]				
Elevated waist circumference	94,296 (61.9)	49,570 (47.4)	44,726 (93.6)	<0.001
Hypertriglyceridemia	45,732 (30.0)	13,796 (13.2)	31,936 (66.9)	<0.001
Elevated blood pressure	82,430 (54.1)	40,561 (38.8)	41,869 (87.7)	<0.001
Hyperglycemia	48,439 (31.8)	17,246 (16.5)	31,193 (65.3)	<0.001
Reduced HDL‐cholesterol	17,002 (11.2)	6820 (6.5)	10,182 (21.3)	<0.001

HDL. High-density lipoprotein; SD. Standard deviation

### 3.2 MetS and risk of sepsis and 28-day mortality following sepsis

During a median follow-up of 13.7 years, 11,040 incident cases of sepsis and 2105 cases of 28-day mortality following sepsis were identified in the UK Biobank from linked hospital inpatient records and death registers, based on predefined ICD-9 and -10 codes. In the Kailuan Study, we identified 5672 incident sepsis cases and 988 cases of 28-day mortality following sepsis during the median follow-up of 16.4 years, and among all sepsis cases, 4619 (81.4%) were identified based on the Sepsis-3 criteria and 1053 (18.6%) were identified using ICD codes (**Additional file 1:** Table S9). Participants with MetS had higher incidence rates of both sepsis and 28-day mortality following sepsis compared with those without MetS in both the UK Biobank and the Kailuan Study (sepsis: 3.70 vs. 1.66 and 3.69 vs. 2.24 per 1000 person-years, respectively; mortality: 0.73 vs. 0.30 and 0.68 vs. 0.36 per 1000 person-years, respectively; Table 3). In the fully adjusted model of the UK Biobank, MetS was associated with a higher risk of sepsis incidence (*HR*=1.55, 95% CI 1.49–1.61, *P*<0.001) and 28-day mortality following sepsis (*HR*=1.51, 95% CI 1.37–1.65, *P*<0.001, Table 3). In the fully adjusted model of the Kailuan Study, MetS was associated with an increased risk of sepsis (*HR*=1.32, 95% CI 1.25–1.40, *P*<0.001) and 28-day mortality following sepsis (*HR*=1.49, 95% CI 1.32–1.69, *P*<0.001) (Table 3).

**Table 3:** Associations between metabolic syndrome (MetS) and risk of sepsis and 28-day mortality following sepsis in the UK Biobank and the Kailuan Study.

**Cohort and outcome**	**No MetS**	**MetS**	***P*****-value**
UK Biobank			
Sepsis incidence			
No. of cases/all participants	5384/242,571	5656/117,062	
Person year	3,248,447	1,526,906	
Incidence rate per 1000 person-years	1.66	3.70	
Model 1^a^ [*HR* (95% CI)]	Reference	1.80 (1.73–1.87)	<0.001
Model 2^b^ [*HR* (95% CI)]	Reference	1.62 (1.56–1.68)	<0.001
Model 3^c^ [*HR* (95% CI)]	Reference	1.55 (1.49–1.61)	<0.001
28-day mortality following sepsis			
No. of cases/all participants	973/242,571	1132/117,062	
Person year	3,263,639	1,541,434	
Incidence rate per 1000 person-years	0.30	0.73	
Model 1^a^ [*HR* (95% CI)]	Reference	1.84 (1.69–2.01)	<0.001
Model 2^b^ [*HR* (95% CI)]	Reference	1.60 (1.47–1.75)	<0.001
Model 3^c^ [*HR* (95% CI)]	Reference	1.51 (1.37–1.65)	<0.001
Kailuan Study			
Sepsis incidence			
No. of cases/all participants	3274/104,554	2398/47,763	
Person year	1,459,976	650,732	
Incidence rate per 1000 person-years	2.24	3.69	
Model 1^d^ [*HR* (95% CI)]	Reference	1.32 (1.25–1.39)	<0.001
Model 2^e^ [*HR* (95% CI)]	Reference	1.32 (1.25–1.40)	<0.001
28-day mortality following sepsis			
No. of cases/all participants	538/104,554	450/47,763	
Person year	1,474,010	660,239	
Incidence rate per 1000 person-years	0.36	0.68	
Model 1^d^ [*HR* (95% CI)]	Reference	1.48 (1.30–1.68)	<0.001
Model 2^e^ [*HR* (95% CI)]	Reference	1.49 (1.32–1.69)	<0.001

a Model 1: adjusted for age and sex.

b Model 2: adjusted for age, sex, ethnicity, educational level, assessment center, Townsend deprivation index, smoking status, alcohol consumption, healthy diet, regular physical activity, and sleep duration.

c Model 3: adjusted for age, sex, ethnicity, educational level, assessment center, Townsend deprivation index, smoking status, alcohol consumption, healthy diet, regular physical activity, sleep duration, aspirin use, non-aspirin non-steroidal anti-inflammatory drug use, vitamin supplementation, and mineral and other dietary supplementation.

d Model 1: adjusted for age and sex.

e Model 2: adjusted for age, sex, educational level, assessment center, smoking status, alcohol consumption, physical activity, and sleep duration. CI. Confidence interval; *HR*. Hazard ratio

### 3.3 Stratified analyses

**Fig. 1:**
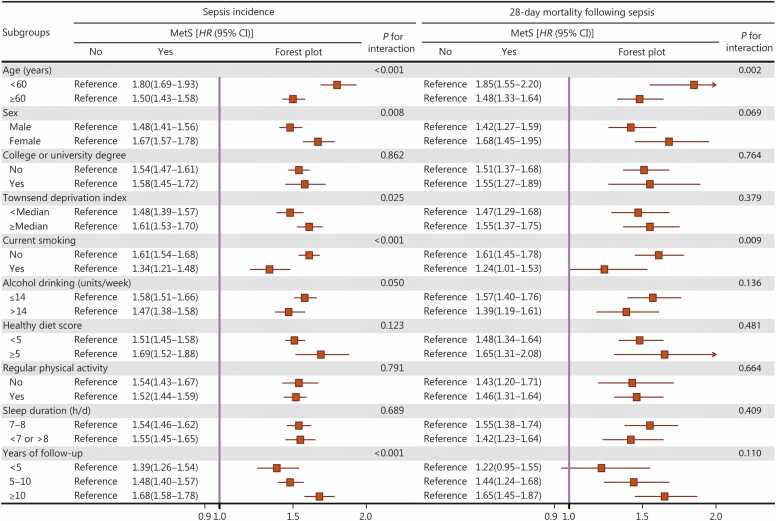
**Stratified analyses for the associations between metabolic syndrome (MetS) and risk of sepsis and 28-day mortality following sepsis in the UK Biobank (*****n*****=359,633).** Multivariable models were adjusted for age, sex, ethnicity, educational level, assessment center, Townsend deprivation index, smoking status, alcohol consumption, healthy diet, regular physical activity, sleep duration, aspirin use, non-aspirin non-steroidal anti-inflammatory drug use, vitamin supplementation, and mineral and other dietary supplementation. CI. Confidence interval; *HR*. Hazard ratio.

In the UK Biobank, stratified analyses demonstrated generally similar results by sex, educational level, TDI, alcohol use, healthy diet, physical activity, and sleep duration (all *P* for interaction >0.005, Fig. 1). On the other hand, a stronger association was observed for sepsis incidence among participants at age <60 years at baseline (*HR*=1.80, 95% CI 1.69–1.93) compared to older participants (*HR*=1.50, 95% CI 1.43–1.58) (*P* for interaction <0.001), among those who were not current smokers (*HR*=1.61, 95% CI 1.54–1.68) compared to current smokers (*HR*=1.34, 95% CI 1.21–1.48) (*P* for interaction <0.001), and among participants with a longer follow-up duration (≥10 years: *HR*=1.68, 95% CI 1.58–1.78) (*P* for interaction <0.001). In terms of 28-day mortality following sepsis, the association appeared to be more pronounced among individuals at age below 60 (*HR*=1.85, 95% CI 1.55–2.20) compared to older individuals (*P* for interaction=0.002). In the Kailuan Study, results from the stratified analyses also remained largely consistent, regardless of sex, education level, smoking, alcohol use, physical activity, or sleep duration (all *P* for interaction >0.005, **Additional file 1:** Fig. S4). However, stronger associations were also observed among younger individuals, particularly for sepsis incidence (*P* for interaction <0.001).

### 3.4 Dose-response associations

In the UK Biobank cohort, all individual components of MetS, including elevated waist circumference, hypertriglyceridemia, elevated blood pressure, hyperglycemia, and reduced HDL-cholesterol, were significantly associated with an increased risk of sepsis, with a 17%–56% risk increment in Model 3 (Table 4). All individual components, with the exception of hypertriglyceridemia, were also associated with an elevated risk of 28-day mortality following sepsis, with a 34%–51% risk increment in Model 3. In the analysis by the number of MetS components, we observed a dose-response relationship between the number of present MetS components and sepsis incidence (Model 3, *HRs*=1.08, 1.25, 1.50, 1.94, and 2.59) as well as 28-day mortality following sepsis (Model 3, *HRs*=1.20, 1.36, 1.66, 2.10, and 2.36) in relation to the presence of one, two, three, four, and five components of MetS, compared to the absence of any MetS component, respectively (all *P* for trend <0.001, Table 4).

**Table 4:** Associations between metabolic syndrome (MetS) components and risk of sepsis and 28-day mortality following sepsis in the UK Biobank [*HR* (95% CI)].

**MetS component**	**Sepsis incidence**			**28-day mortality following sepsis**		
	Model 1^a^	Model 2^b^	Model 3^c^	Model 1^a^	Model 2^b^	Model 3^c^
Elevated waist circumference						
No	Reference	Reference	Reference	Reference	Reference	Reference
Yes	1.75 (1.68–1.82)	1.61 (1.55–1.67)	1.56 (1.50–1.62)	1.65 (1.51–1.80)	1.48 (1.35–1.61)	1.42 (1.30–1.56)
Hypertriglyceridemia						
No	Reference	Reference	Reference	Reference	Reference	Reference
Yes	1.24 (1.20–1.29)	1.17 (1.13–1.21)	1.17 (1.13–1.21)	1.15 (1.06–1.26)	1.07 (0.98–1.16)	1.07 (0.98–1.16)
Elevated blood pressure						
No	Reference	Reference	Reference	Reference	Reference	Reference
Yes	1.20 (1.15–1.27)	1.20 (1.14–1.26)	1.17 (1.11–1.23)	1.39 (1.23–1.58)	1.38 (1.22–1.57)	1.34 (1.18–1.51)
Hyperglycemia						
No	Reference	Reference	Reference	Reference	Reference	Reference
Yes	1.79 (1.72–1.86)	1.60 (1.54–1.67)	1.52 (1.46–1.59)	1.87 (1.71–2.04)	1.61 (1.47–1.77)	1.51 (1.38–1.66)
Reduced HDL-cholesterol						
No	Reference	Reference	Reference	Reference	Reference	Reference
Yes	1.69 (1.63–1.75)	1.53 (1.47–1.59)	1.43 (1.37–1.49)	1.74 (1.60–1.90)	1.53 (1.40–1.68)	1.40 (1.27–1.53)
Number of MetS components						
0	Reference	Reference	Reference	Reference	Reference	Reference
1	1.10 (1.01–1.19)	1.08 (0.99–1.17)	1.08 (0.99–1.17)	1.24 (1.00–1.54)	1.20 (0.97–1.50)	1.20 (0.97–1.50)
2	1.33 (1.23–1.45)	1.26 (1.16–1.37)	1.25 (1.14–1.35)	1.51 (1.22–1.87)	1.39 (1.12–1.73)	1.36 (1.09–1.69)
3	1.72 (1.58–1.87)	1.55 (1.42–1.69)	1.50 (1.38–1.64)	2.01 (1.62–2.49)	1.74 (1.40–2.16)	1.66 (1.34–2.06)
4	2.37 (2.18–2.59)	2.04 (1.87–2.23)	1.94 (1.78–2.12)	2.80 (2.25–3.49)	2.27 (1.82–2.83)	2.10 (1.68–2.63)
5	3.42 (3.11–3.76)	2.80 (2.54–3.08)	2.59 (2.35–2.85)	3.48 (2.74–4.43)	2.64 (2.07–3.37)	2.36 (1.85–3.02)
*P* for trend	<0.001	<0.001	<0.001	<0.001	<0.001	<0.001

a Model 1: adjusted for age and sex.

b Model 2: further adjusted for ethnicity, educational level, assessment center, Townsend deprivation index, smoking status, alcohol consumption, healthy diet, regular physical activity, and sleep duration.

c Model 3: further adjusted for aspirin use, non-aspirin non-steroidal anti-inflammatory drug use, vitamin supplementation, and mineral and other dietary supplementation. CI. Confidence interval; HDL. High-density lipoprotein; SD. Standard deviation; *HR*. Hazard ratio

### 3.5 Mediation analyses

Mediation analyses revealed that 22.5% (95% CI 19.9–25.5), 11.0% (95% CI 9.1–13.3), 8.1% (95% CI 6.6–9.8), 4.6% (95% CI 3.5–6.2), and 3.6% (95% CI 2.8–4.5) of the association between MetS and sepsis incidence, and 25.1% (95% CI 18.8–32.7), 14.5% (95% CI 9.9–20.8), 12.8% (95% CI 9.1–17.7), 6.0% (95% CI 3.4–10.3), and 6.2% (95% CI 4.1–9.1) of the association between MetS and 28-day mortality following sepsis could be mediated through the blood CRP level, white blood cell count, neutrophil count, monocyte count, and IGF-1 level, respectively (all *P*<0.001, Table 5).

**Table 5:** Blood biomarkers as mediators of the association between metabolic syndrome (MetS) and risk of sepsis and 28-day mortality following sepsis in the UK Biobank^a^.

**Blood marker**	**Sepsis incidence**				**28-day mortality following sepsis**			
	**Unadjusted for the blood marker [*****HR*****(95% CI)]**	**Adjusted for the blood marker [*****HR*****(95% CI)]**	**Mediation proportion [% (95% CI)]**	***P*****for mediation**	**Unadjusted for the blood marker [*****HR*****(95% CI)]**	**Adjusted for the blood marker [*****HR*****(95% CI)]**	**Mediation proportion [% (95% CI)]**	***P*****for mediation**
CRP (mg/L)	1.55 (1.49–1.61)	1.40 (1.35–1.46)	22.5 (19.9–25.5)	<0.001	1.50 (1.37–1.65)	1.36 (1.24–1.49)	25.1 (18.8–32.7)	<0.001
White blood cell count (×10^9^/L)	1.55 (1.49–1.61)	1.48 (1.42–1.54)	11.0 (9.1–13.3)	<0.001	1.51 (1.38–1.66)	1.42 (1.30–1.56)	14.5 (9.9–20.8)	<0.001
Neutrophil count (×10^9^/L)	1.55 (1.49–1.61)	1.50 (1.44–1.56)	8.1 (6.6–9.8)	<0.001	1.51 (1.38–1.66)	1.43 (1.31–1.57)	12.8 (9.1–17.7)	<0.001
Monocyte count (×10^9^/L)	1.55 (1.49–1.61)	1.52 (1.46–1.58)	4.6 (3.5–6.2)	<0.001	1.51 (1.38–1.66)	1.48 (1.35–1.62)	6.0 (3.4–10.3)	<0.001
PLR	1.55 (1.49–1.61)	1.56 (1.50–1.63)	None	-	1.51 (1.38–1.66)	1.55 (1.41–1.70)	None	-
SII^b^	1.55 (1.49–1.61)	1.55 (1.49–1.61)	None	-	1.51 (1.38–1.66)	1.52 (1.39–1.67)	None	-
Lymphocyte count (×10^9^/L)	1.55 (1.49–1.61)	1.57 (1.51–1.64)	None	-	1.51 (1.38–1.66)	1.58 (1.44–1.73)	None	-
IGF-1 (nmol/L)	1.55 (1.49–1.61)	1.52 (1.46–1.59)	3.6 (2.8–4.5)	<0.001	1.50 (1.37–1.64)	1.46 (1.33–1.60)	6.2 (4.1–9.1)	<0.001

^b^Systemic immune-inflammation index (SII)=neutrophil×platelet/lymphocyte. CI. Confidence interval; CRP. C-reactive protein; *HR*. Hazard ratio; IGF-1. Insulin-like growth factor-1; PLR. Platelet-to-lymphocyte ratio

a Multivariable model was adjusted for age, sex, ethnicity, educational level, assessment center, Townsend deprivation index, smoking status, alcohol consumption, healthy diet, regular physical activity, sleep duration, aspirin use, non-aspirin non-steroidal anti-inflammatory drug use, vitamin supplementation, and mineral and other dietary supplementation.

### 3.6 Joint association of MetS and lifestyle with sepsis outcomes

Compared to individuals free of MetS, individuals with MetS and an unfavorable lifestyle had a 91% increased risk of sepsis (*HR*=1.91, 95% CI 1.81–2.00) and 84% increased risk of 28-day mortality following sepsis (*HR*=1.84, 95% CI 1.64–2.07) (Table 6). Individuals with MetS and an intermediate lifestyle had a 46% increased risk of sepsis (*HR*=1.46, 95% CI 1.37–1.55) and 46% increased risk of 28-day mortality following sepsis (*HR*=1.46, 95% CI 1.28–1.68). Although individuals with MetS and a favorable lifestyle showed an 18% increased risk of sepsis (*HR*=1.18, 95% CI 1.09–1.28), they showed no excess risk of 28-day mortality following sepsis (*HR*=1.05, 95% CI 0.88–1.27). Additionally, MetS was associated with an increased risk of 7-day, 60-day, 90-day, 180-day, and 1-year mortality following sepsis, representing a 49%–54% increased risk in Model 3 (**Additional file 1:** Table S10).

**Table 6:** Potential joint effect of metabolic syndrome (MetS) and lifestyle on risk of sepsis and 28-day mortality following sepsis in the UK Biobank^a^, ^b^.

**Groups**	**Sepsis incidence**		**28-day mortality following sepsis**	
	***HR*****(95% CI)**	***P*****-value**	***HR*****(95% CI)**	***P*****-value**
Individuals free of MetS	Reference		Reference	
Individuals with MetS				
Favorable lifestyle	1.18 (1.09–1.28)	<0.001	1.05 (0.88–1.27)	0.584
Intermediate lifestyle	1.46 (1.37–1.55)	<0.001	1.46 (1.28–1.68)	<0.001
Unfavorable lifestyle	1.91 (1.81–2.00)	<0.001	1.84 (1.64–2.07)	<0.001

a Adjusted for age, sex, ethnicity, educational level, assessment center, Townsend deprivation index, aspirin use, non-aspirin non-steroidal anti-inflammatory drug use, vitamin supplementation, and mineral and other dietary supplementation.

b Unhealthy lifestyle score was generated based on: 1) obesity (score of 1: body mass index ≥30 kg/m²); 2) current smoking (score of 1); 3) excessive alcohol consumption (score of 1: >14 units/week); 4) unhealthy diet (score of 1: healthy diet score <5); 5) insufficient physical activity (score of 1: not meeting the criterium of ≥150 min of moderate activity per week, ≥75 min of vigorous activity per week, an equivalent combination, moderate activity on ≥5 d per week, or vigorous activity at least once per week); and 6) suboptimal sleep duration (score of 1: <7 or >8 h per day), ranging from 0–6 and with a higher score indicating an unhealthier lifestyle. The study participants were then categorized into three groups based on tertiles of the score: favorable (score <2), intermediate (score =2), and unfavorable (score >2) lifestyle. CI. Confidence interval; *HR*. Hazard ratio

### 3.7 Associations of MetS evolution with risk of sepsis

In the sub-cohort of the Kailuan Study, including 62,578 participants with data from both the 2006 and 2010 examinations (**Additional file 1:** Table S11), participants who recovered from MetS did not show an increased risk of sepsis (*HR*=1.09, 95% CI 0.96–1.25), whereas those with MetS progression (*HR*=1.17, 95% CI 1.05–1.31) or persistent MetS (*HR*=1.46, 95% CI 1.32–1.61) demonstrated significantly increased risk of sepsis, compared to participants with persistently normal metabolic status (*P* for trend <0.001, Table 7). The corresponding *HR*s were 1.19 (95% CI 0.87–1.61), 1.36 (95% CI 1.04–1.77), and 1.88 (95% CI 1.50–2.35), respectively, for 28-day mortality following sepsis (*P* for trend <0.001, Table 7).

**Table 7:** Associations between metabolic syndrome (MetS) evolution and risk of sepsis and 28-day mortality following sepsis in a sub-cohort of the Kailuan Study.

**Outcome and model**	**MetS evolution group**				***P*****for trend**
	**Sustained metabolic health**	**MetS recovery**	**MetS progression**	**Persistent MetS**	
Sepsis incidence					
No. of cases/all participants	1005/31,827	303/6924	445/11,057	697/12,770	
Model 1^a^ [*HR* (95% CI)]	Reference	1.09 (0.96–1.24)	1.19 (1.07–1.33)	1.46 (1.32–1.61)	<0.001
Model 2^b^ [*HR* (95% CI)]	Reference	1.09 (0.96–1.25)	1.17 (1.05–1.31)	1.46 (1.32–1.61)	<0.001
28-day mortality following sepsis					
No. of cases/all participants	166/31,827	56/6924	84/11,057	148/12,770	
Model 1^a^ [*HR* (95% CI)]	Reference	1.17 (0.87–1.59)	1.37 (1.05–1.78)	1.85 (1.48–2.31)	<0.001
Model 2^b^ [*HR* (95% CI)]	Reference	1.19 (0.87–1.61)	1.36 (1.04–1.77)	1.88 (1.50–2.35)	<0.001

a Model 1: adjusted for age and sex.

b Model 2: further adjusted for educational level, assessment center, smoking status, alcohol consumption, physical activity, and sleep duration. CI. Confidence interval; *HR*. Hazard ratio

### 3.8 Sensitivity analyses

In the sensitivity analyses, the associations remained largely unchanged after excluding the first two years of follow-up, excluding participants with missing data on covariates, using a competing risk model, redefining MetS using the 2005 IDF criteria, or using multiple imputation to impute missing data in the UK Biobank and the Kailuan Study (**Additional file 1:** Tables S12, S13). In the UK Biobank, excluding participants who had a fasting time of less than 3 h before blood sampling, further adjusting for major comorbidities at baseline, or using an alternative method based on the Sepsis-3 definition to ascertain sepsis cases, did not lead to different results either. Similarly, excluding BMI from the lifestyle score did not change the results of the analyses regarding the joint effect of MetS and lifestyle on sepsis risk and the 28-day mortality following sepsis (**Additional file 1:** Table S14). Sepsis risk varied markedly across the 16 MetS phenotypes. For example, the phenotype comprising 4 components except reduced HDL-cholesterol or hypertriglyceridemia showed a particular strong association with incident sepsis (except reduced HDL-cholesterol: *HR*=1.65, 95% CI 1.47–1.86; except hypertriglyceridemia: *HR*=2.17,  95%  CI 1.98–2.39) and 28‑day mortality following sepsis (except reduced HDL-cholesterol: *HR*=2.10, 95% CI 1.67–2.65; except hypertriglyceridemia: *HR*=1.91,  95% CI 1.55–2.36; **Additional file 1:** Table S15).

## 4 Discussion

In this study, including 359,633 participants from the UK Biobank and 152,317 participants from the Kailuan Study, we found that MetS was associated with an increased risk of sepsis incidence and 28-day mortality following sepsis. Specifically, there was a 159% and 136% risk increment for sepsis and 28-day mortality following sepsis among individuals who had all 5 studied components of MetS. Notably, these associations were independent of traditional risk factors for sepsis and remained consistent across most subgroups. Furthermore, mediation analysis elucidated that blood inflammatory biomarkers, including CRP, white blood cell count, and neutrophil count, partly mediated the association between MetS and sepsis outcomes. We also found that individuals with both MetS and an unfavorable lifestyle had the highest risk of sepsis and 28-day mortality following sepsis. Individuals with MetS but a favorable lifestyle showed a substantially attenuated risk of sepsis, with no excess risk of 28-day mortality following sepsis. Finally, in a sub-sample of the Kailuan Study, we found that participants with persistent MetS had a higher risk of sepsis and 28-day mortality following sepsis, whereas those who recovered from MetS showed no excess risk of these outcomes, compared with participants who remained metabolically healthy.

This study investigated the associations between MetS and risk of sepsis and sepsis-related mortality. Previous large-scale prospective studies have demonstrated considerable associations of MetS with an increased risk of multiple chronic diseases, including dementia, anxiety disorders, mortality, and cancer [23], [40], [41], [42]. However, epidemiological studies examining the link between MetS and sepsis remain sparse. A previous cohort study of over 120,000 participants reported that MetS status was associated with a 97% elevated risk of severe COVID-19, and a 74% increased mortality risk among COVID-19 patients who had MetS [43]. Additionally, a population-based cohort study with over 30,000 participants and a follow-up of over 8 years demonstrated that morbid obesity (BMI >40 kg/m^2^) and an increased waist circumference were associated with a 57% and 34% increased risk of sepsis, respectively, after multivariable adjustment [44]. The findings in this study expand the existing knowledge base by demonstrating that the combination of multiple abnormal metabolic components, namely MetS, is significantly associated with an increased risk of sepsis and sepsis-related mortality. Notably, we observed stronger associations with sepsis among younger participants. This pattern likely reflects the substantially lower absolute risk of sepsis in younger individuals: when the absolute risk is low, MetS may play a relatively more important role, leading to a greater relative risk. In contrast, in older adults with higher absolute sepsis risk, the incremental contribution of MetS might appear smaller on a relative scale. This study also demonstrates a dose-dependent association between the severity of MetS (indicated by the number of present MetS components) and the risk of sepsis as well as 28-day mortality following sepsis. The component-level analyses revealed that dysregulated glucose homeostasis, elevated blood pressure, and elevated waist circumference played an important role in the development and progression of sepsis. Hyperglycemia and hypertension are known to impair innate immune function, disrupt endothelial integrity, and exacerbate microvascular dysfunction, all of which are key pathophysiological processes underlying the development and progression of sepsis [45], [46]. Similarly, elevated waist circumference, a marker of central adiposity, was a key component in the MetS phenotypes associated with increased sepsis risk, which is in line with prior studies demonstrating an independent role of central adiposity in sepsis [44], [47]. However, previous studies have demonstrated that the association between triglyceride levels and mortality, including sepsis-related mortality, commonly shows a U-shaped pattern [48], [49]. Therefore, dichotomizing triglyceride levels using the MetS threshold may distribute high-risk individuals in both categories (e.g., the increased risk in relation to very low triglyceride levels is reflected in the reference group), leading to a potential attenuation of the observed association for the hypertriglyceridemia component.

Further, the findings in this study demonstrate that MetS might interact with an unhealthy lifestyle in modulating the risk of sepsis and sepsis-related mortality, because individuals with MetS and an unhealthy lifestyle experienced an almost double risk of sepsis and sepsis-related mortality, whereas those with MetS and a healthy lifestyle showed an 18% higher risk of sepsis and no increased risk of sepsis-related mortality. These observations highlight that a favorable lifestyle, such as regular physical activity, non-smoking, healthy diet, and healthy sleep patterns, may serve as a potent modifier of the metabolism-sepsis axis [50], [51], [52]. This is consistent with the hypothesis that such healthy behaviors enhance immunometabolic regulation and attenuate the chronic low-grade systemic inflammation characteristic of MetS, thereby influencing both the onset and progression of sepsis [53], [54]. Lifestyle factors can influence sepsis risk both directly and indirectly through modulating metabolic status. For example, sleep duration and quality may influence sepsis risk directly by modulating immune and inflammatory responses. In a large population-based prospective cohort, individuals with unhealthy sleep patterns exhibited a significantly increased risk of developing sepsis [51]. Experimental evidence further supports this mechanism, showing that sleep disruption epigenetically reprograms hematopoietic stem and progenitor cells, promotes myeloid skewing, and amplifies inflammatory responses upon subsequent infectious challenges, ultimately worsening sepsis outcomes [55]. Finally, by incorporating repeated metabolic assessments, the findings in this study also demonstrate that individuals who progress to or maintain MetS may be at substantially increased risk of sepsis and 28-day mortality following sepsis, whereas those who recover from MetS may not have excess risk compared with individuals who remain metabolically healthy. Given the role of a healthy lifestyle in sepsis [51], [56], the lower risk observed in the “MetS recovery” group may reflect both improved metabolic status and a healthier lifestyle, whereas the “MetS progression” and “persistent MetS” groups likely experienced adverse trajectories leading to higher sepsis risk.

The mediation analyses identified inflammatory pathways as key biological mechanisms mediating the association between MetS and sepsis risk. This is consistent with a previous study showing that MetS and its individual components contribute to a persistent, low-grade inflammatory state, commonly referred to as “metaflammation” [57], [58], [59]. This low-grade inflammation disrupts immune homeostasis and impairs immunoregulation during infection, thereby increasing the risk of sepsis onset and progression [60]. For example, in metabolically abnormal individuals, adipose tissue macrophages undergo a phenotypic shift from an anti-inflammatory M2 state to a proinflammatory M1 state, resulting in substantial M1 infiltration, persistent cytokine release, and the development of an inflammatory micro-environment [61], [62], [63]. In addition, the increased metabolic load of MetS may contribute to endoplasmic reticulum stress, which may activate the unfolded protein response and enhance the expression of inflammatory mediators via the c-Jun N-terminal kinase (JNK) and nuclear factor κB (NF-κB) signaling pathways [64], [65]. MetS-related metabolic dysregulation is characterized by a significant reduction in short-chain fatty acids-producing bacteria, accompanied by an increased abundance of Gram-negative bacteria rich in lipopolysaccharide (LPS) [66], [67], [68]. The translocation of LPS into the circulation may induce metabolic endotoxemia, subsequently leading to sustained inflammatory responses via the Toll-like receptor 4 (TLR4) signaling pathway [69], [70], [71], [72]. As a result, persistent inflammatory stimuli may continuously activate innate immune signaling pathways and progressively disrupt immune homeostasis [73], [74], impairing the body’s capacity to eliminate pathogens, triggering detrimental immune responses, exacerbating tissue damage, and ultimately contributing to the onset and progression of sepsis [75].

MetS may aid sepsis risk stratification, as individuals with clustered metabolic abnormalities appear more prone to progress from infection to life-threatening organ dysfunction and to experience worse outcomes following sepsis. These results support a conceptual shift, namely that risk of sepsis is shaped not only at the time of infection but also by a pre-existing host milieu in which infection acts as a trigger. The results of this study support a link between metabolic dysregulation and immune-mediated adverse outcomes. They also align with emerging applications of advanced metabolic profiling, such as continuous glucose monitoring-based machine learning and integrative genetic-glucose analyses, which have improved prediction of immune-related complications in MetS-related conditions, including diabetes [76], [77].

The primary strengths of this study include its large sample size from two prospective cohorts with up to 17 years of follow-up, diverse ethnic representation, and comprehensive data on socioeconomic characteristics, lifestyle factors, medical history, and other relevant covariates. Meanwhile, several limitations need to be considered. First, the status of MetS was determined slightly differently between cohorts. For example, as the UK Biobank predominantly includes individuals of European ancestry, we applied waist circumference thresholds of ≥102 cm for men and ≥88 cm for women. In contrast, as the Kailuan Study includes only the Chinese population, we applied waist circumference thresholds of ≥85 cm for men and ≥80 cm for women, in accordance with guideline recommendations in China. Further, sepsis was identified from hospital and death registries using ICD codes in the UK Biobank, whereas in the Kailuan Study, we identified sepsis cases through detailed clinical data according to the Sepsis-3 criteria as well as hospital records using ICD codes. Although heterogeneity between the two cohorts may limit comparability in the findings, incorporating diverse populations provides an opportunity for cross-validation and substantiate generalizability of the findings. Moreover, the consistent findings noted between the two cohorts of largely different ethnicity and healthcare systems also argue against residual confounding from these factors as an important contributor to the study results. Second, residual confounding from unknown or unmeasured factors cannot be entirely ruled out. As noted above, because of substantial differences in data availability between the two cohorts, we were unable to adjust for an identical set of covariates between the datasets. For example, the UK Biobank has more detailed information on socioeconomic factors and medications/supplement use, including TDI, diet, and use of anti-inflammatory medications and supplements, whereas no such data were available in the Kailuan Study. Accordingly, these covariates were adjusted for in the analyses of the UK Biobank only. Nonetheless, the associations remained robust after adjustment for a broad range of potential confounding factors and remained consistent across multiple stratified analyses in both cohorts. Third, we need to acknowledge that sepsis ascertainment differed across cohorts, with an ICD-based algorithm used in the UK Biobank and a Sepsis-3/SOFA-based (in combination with ICD codes) algorithm in the Kailuan Study. To minimize bias from non-identical definitions, we analyzed the cohorts as independent study samples and emphasized concordance in the direction and magnitude of associations rather than pooling individual-level data for analysis. We further evaluated the robustness of the findings using alternative outcome definitions (including ICD-based definitions with greater sensitivity/definition of severe sepsis). However, any outcome misclassification is expected to be likely non-differential with respect to baseline exposure status, which would tend to attenuate the results toward the null. Moreover, MetS status was assessed only at recruitment in the UK Biobank and might have changed during follow-up. Nonetheless, we were able to examine MetS evolution in the Kailuan Study to partially address this concern. Last but not least, the study cohorts are rather different in terms of ethnicity and sociocultural context, e.g., participants of the UK Biobank are predominantly White European descent and were recruited from the general community in the UK, whereas participants of the Kailuan Study are exclusively Chinese coal miners. Substantial differences also exist in lifestyle, health status, and healthcare use between the participants of the two cohorts, in addition to the different ascertainment methods of exposures and outcomes. Instead of pooling individual-level data together in a manner that could obscure heterogeneity, we treated the two cohorts as independent study samples and focused on identifying consistency in the direction and magnitude of associations between the two cohorts. Indeed, we observed consistent direction and broadly similar magnitude of the studied associations between the two cohorts. This provides a validation across divergent populations and ascertainment frameworks, thereby substantiating the robustness and external generalizability of the overall conclusions.

## 5 Conclusions

This study, combining data from the UK Biobank and the Kailuan Study, demonstrates that MetS is associated with an increased risk of sepsis and sepsis-related mortality. The excess risk increases progressively with the number of MetS components, and is partly mediated through inflammatory biomarkers. Moreover, adherence to a favorable lifestyle substantially mitigates the excess risk associated with MetS. These findings provide compelling evidence to support the potential role of MetS in the etiology of sepsis and highlight the importance of maintaining metabolic health and promoting healthy lifestyles as potential strategies to reduce the burden of sepsis.

## Abbreviations

AHA: American Heart Association

BMI: Body mass index

CIs: Confidence intervals

CRP: C-reactive protein

HbA1c: Glycated hemoglobin A1c

HDL: High-density lipoprotein

*HR*: Hazard ratio

ICD: International Classification of Diseases

IDF: International Diabetes Federation

IGF-1: Insulin-like growth factor-1

JNK: c-Jun N-terminal kinase

LPS: Lipopolysaccharide

MetS: Metabolic syndrome

NF-κB: Nuclear factor κB

NHLBI: National Heart, Lung, and Blood Institute

NSAIDs: Non-steroidal anti-inflammatory drugs

PLR: Platelet-to-lymphocyte ratio

SD: Standard deviation

Sepsis-3: Third International Consensus Definitions for Sepsis and Septic Shock

SII: Systemic immune-inflammation index

SOFA: Sequential Organ Failure Assessment

TDI: Townsend deprivation index

TLR4: Toll-like receptor 4

## Ethics approval and consent to participate

The UK Biobank was constructed under ethical approval obtained by the North West Multi-Centre Research Ethics Committee (11/NW/03820), and all participants provided written informed consent prior to participation. The Kailuan Study protocol was approved by the Ethics Committee of Kailuan General Hospital (2006-05), and written informed consent was obtained from all participants.

## Funding

This work was supported by the National Key R&D Program of China (2024YFF1207100), the Fundamental and Interdisciplinary Disciplines Breakthrough Plan of the Ministry of Education of China (JYB2025XDXM506), the Major Program for Fundamental Research of Hunan Provincial (2025JC0002), the National Outstanding Youth Science Fund Project of the National Natural Science Foundation of China (82025021), the Scientific Research Program of FuRong Laboratory (2024PT5103 and 2024PT5105), the National Natural Science Foundation of China (82470931), the Hunan Provincial Excellent Youth Science Fund Project (2024JJ4086), and the Central South University Research Programme of Advanced Interdisciplinary Studies (2023QYJC008).

## Data Availability

Data from the UK Biobank are available to all researchers upon submitting an application. This research was performed using the UK Biobank Resource under Application 98583.

## Footnotes

Appendix A Supplementary data associated with this article can be found in the online version at doi:10.1016/j.mmr.2026.100031.
