# Supplementary material for: Metabolic syndrome and risk of sepsis and sepsis-related mortality: evidence from two large prospective cohort studies
Source: Mil Med Res. 2026 Apr 27;13(1):100031. doi: 10.1016/j.mmr.2026.100031 (PMC13138178; doi:10.1016/j.mmr.2026.100031)
Supplement: Supplementary file 1 — Supplementary material [file mmc1.pdf]

Methods

Definition of unhealthy lifestyle score

In the UK Biobank, participants received 1 point for each of the following risk factors: obesity [body mass index (BMI) ≥30 kg/m²], current smoking, excessive alcohol consumption (>14 units/week), unhealthy diet (healthy diet score <5), insufficient physical activity (not meeting the criterium of ≥150 min of moderate activity per week, ≥75 min of vigorous activity per week, an equivalent combination, moderate activity ≥5 d per week, or vigorous activity at least once per week), and suboptimal sleep duration (< 7 or > 8 h/d). The sum of these scores was used to calculate an unhealthy lifestyle score, ranging from 0–6, with a higher score indicating an unhealthier lifestyle.

ICD-based sepsis ascertainment in the UK Biobank and the Kailuan Study

In the UK Biobank, sepsis events were identified using the 9th and the 10th revisions of the International Classification of Diseases (ICD-9 and -10) codes from linked hospital inpatient and death registry records, and were defined as hospitalizations or death records with a sepsis-related ICD code appearing in any diagnostic position. Only the first qualifying sepsis event during follow-up was included in the analysis. The complete list of ICD-9 and -10 codes used for sepsis identification in the UK Biobank is provided in **Additional file 1: Table S3**.

In the Kailuan Study, ICD-based sepsis ascertainment was applied to capture additional cases. Sepsis events were identified using ICD-9 and -10 codes from hospital discharge diagnoses. Explicit sepsis was defined by the presence of ICD codes explicitly indicating a diagnosis of sepsis or septic shock, whereas implicit sepsis was defined by the co-occurrence of infection-related and acute organ dysfunction ICD codes recorded during the same hospitalization. The corresponding ICD code lists for infection and organ dysfunction are shown in **Additional file 1: Tables S4, S5**.

Sensitivity analyses

To test the robustness of the findings, we conducted several sensitivity analyses in the UK Biobank: 1) to decrease the potential influence of reverse causation, the first two years of follow-up were excluded from the analyses; 2) to reduce the potential influence of missingness, we excluded participants with incomplete data on covariates from the analyses; 3) we also used a multiple imputation approach to impute the missing data; 4) to reduce potential bias arising from competing risk of death, we used a competing risk model and treated death unrelated to sepsis as a competing event; 5) we additionally defined metabolic syndrome (MetS) using the 2005 International Diabetes Federation criteria [1], a widely used alternative case definition; 6) we additionally adjusted the history of cardiovascular disease (CVD), chronic kidney disease (CKD), chronic liver disease (CLD), and chronic obstructive pulmonary disease (COPD), which may act as either a confounder or a mediator of the studied association [2]; 7) to mitigate the impact of fasting duration on metabolic biomarkers, individuals who had fasted for less than 3 h before blood sampling were excluded from the analyses; 8) to improve the sensitivity of sepsis definition, we used an alternative method to ascertain sepsis cases in accordance with the Sepsis-3 definition [3]. Specifically, individuals were also identified as sepsis cases if they had a diagnosis of infection and a diagnosis of acute organ dysfunction documented on the same day [4]; and 9) given the correlation between BMI and MetS, we conducted a sensitivity analysis after excluding BMI from the lifestyle score to assess the joint effect of MetS and lifestyle on sepsis risk.

In the Kailuan Study, sensitivity analyses were conducted using the first five approaches described above (i.e., excluding the first two years of follow-up, excluding participants with missing covariate data, applying multiple imputation for missing data, using non-sepsis death as a competing event, and using the 2005 International Diabetes Federation criteria to define MetS).

References

1. Alberti KG, Zimmet P, Shaw J, Group IDFETFC. The metabolic syndrome--a new worldwide definition. Lancet. 2005;366(9491):1059-62.
2. Qureshi D, Collister J, Allen NE, Kuzma E, Littlejohns T. Association between metabolic syndrome and risk of incident dementia in UK Biobank. Alzheimers Dement. 2024;20(1):447-58.
3. Singer M, Deutschman CS, Seymour CW, Shankar-Hari M, Annane D, Bauer M, *et al.* The Third International Consensus Definitions for Sepsis and Septic Shock (Sepsis-3). JAMA. 2016;315(8):801-10.
4. Garland A, Li N, Sligl W, Lane A, Thavorn K, Wilcox ME, *et al*. Adjudication of codes for identifying sepsis in hospital administrative data by expert consensus. Crit Care Med. 2024;52(12):1845-55.

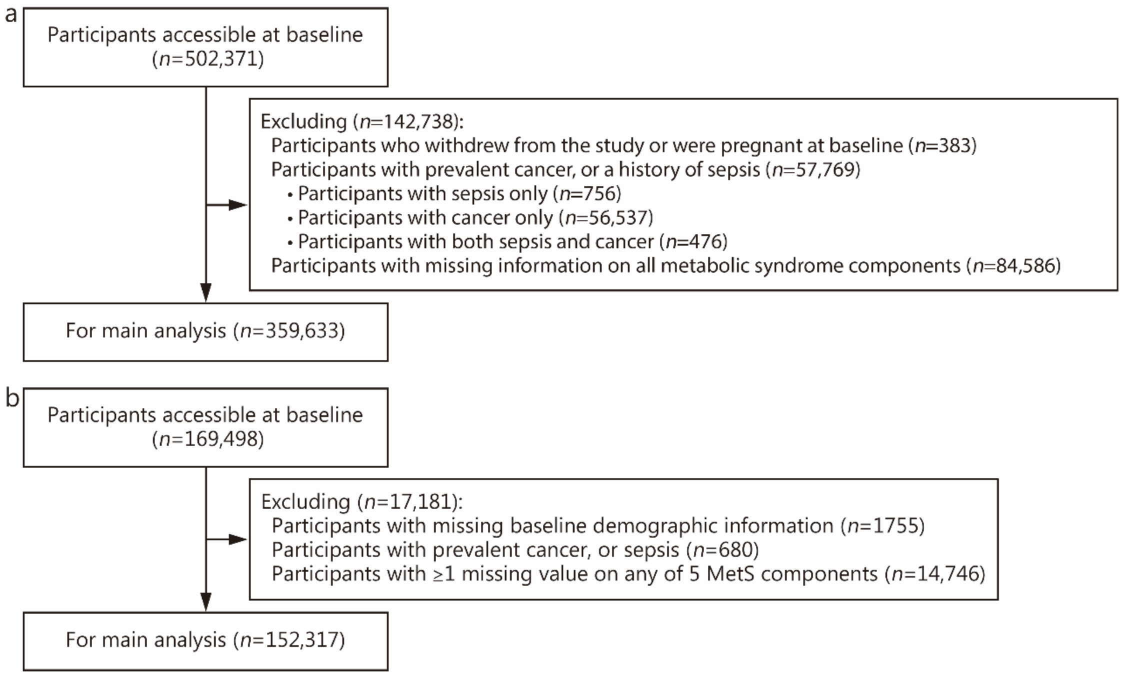

Fig. S1 Flow chart of the study population in the UK Biobank (**a**) and the Kailuan Study (**b**). MetS. Metabolic syndrome

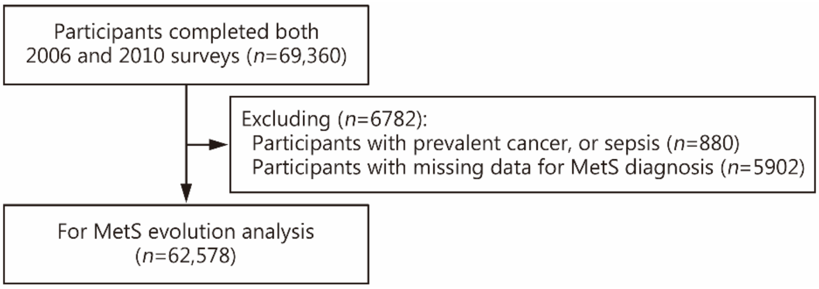

Fig. S2 Flow chart of MetS evolution analysis population in the Kailuan Study. MetS. Metabolic syndrome

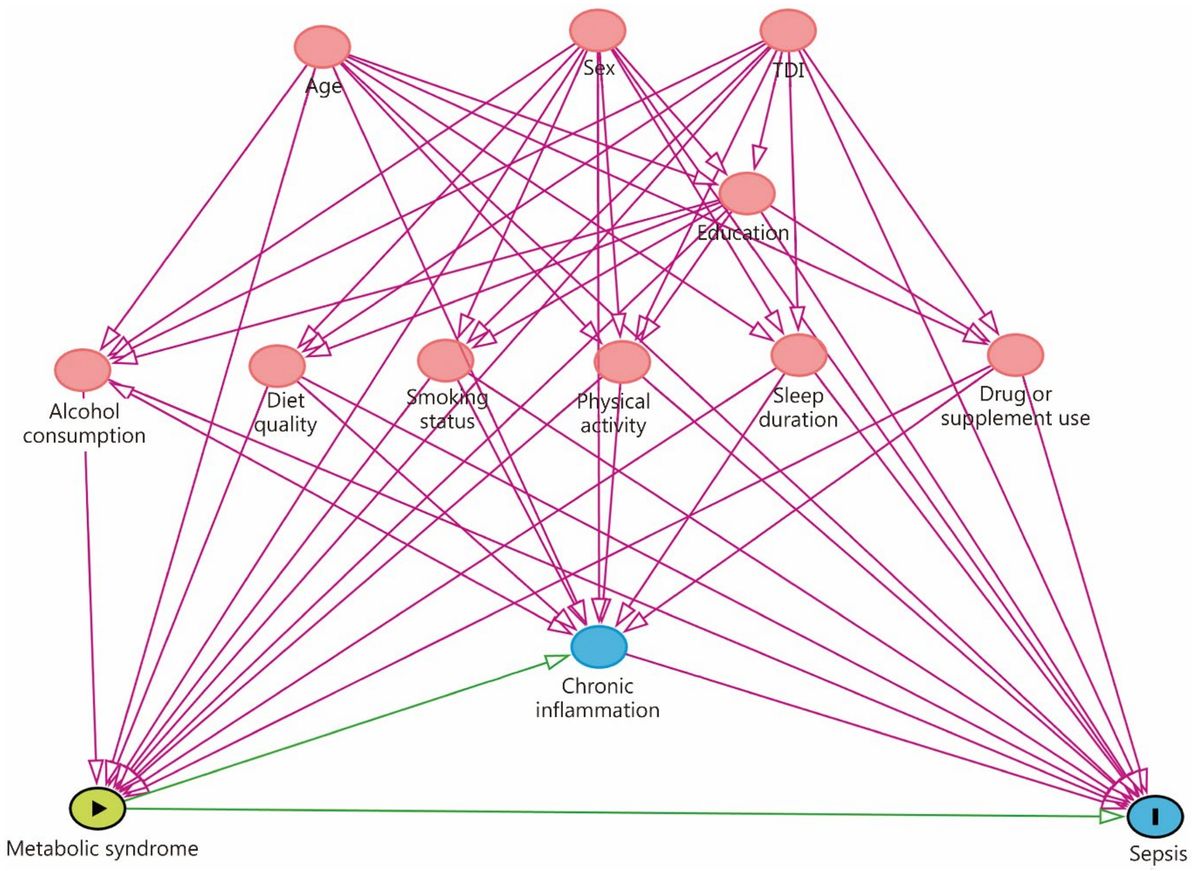

Fig. S3 Directed acyclic graph of the association between metabolic syndrome and risk of sepsis. TDI. Townsend deprivation index

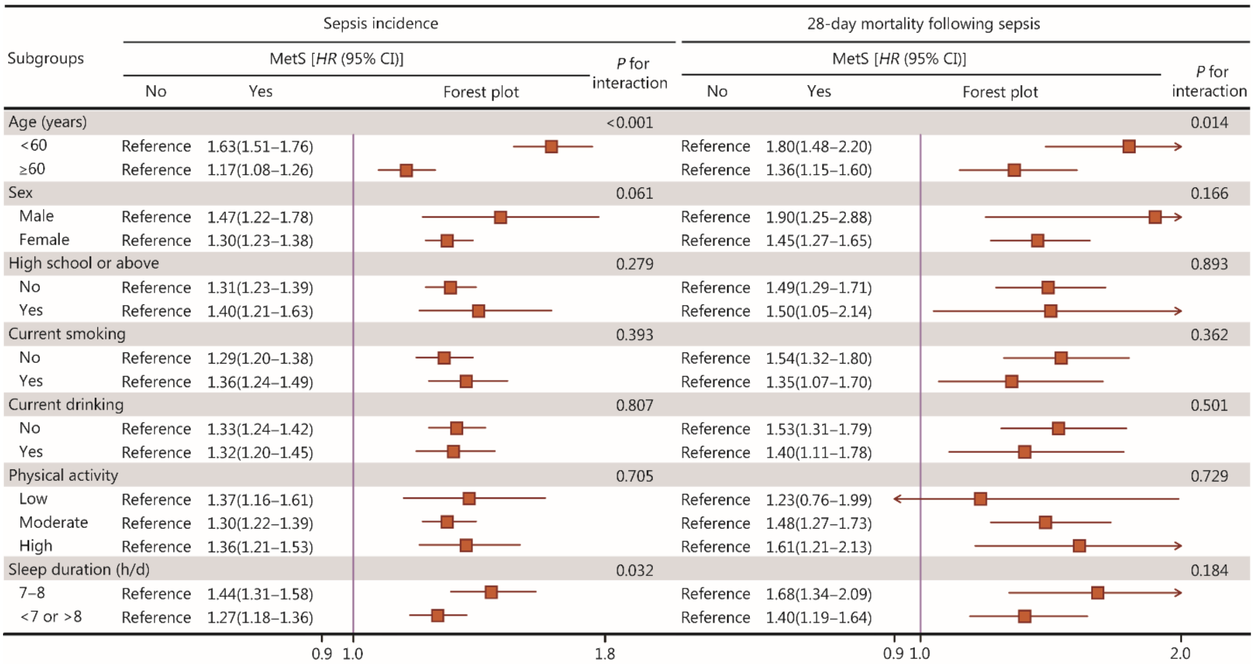

Subgroups	Sepsis incidence: MetS [*HR* (95% CI)] No	Yes	*P* for interaction	28-day mortality following sepsis: MetS [*HR* (95% CI)] No	Yes	*P* for interaction
Age (years)			<0.001			0.014
<60	Reference	1.63(1.51–1.76)		Reference	1.80(1.48–2.20)	
≥60	Reference	1.17(1.08–1.26)		Reference	1.36(1.15–1.60)	
Sex			0.061			0.166
Male	Reference	1.47(1.22–1.78)		Reference	1.90(1.25–2.88)	
Female	Reference	1.30(1.23–1.38)		Reference	1.45(1.27–1.65)	
High school or above			0.279			0.893
No	Reference	1.31(1.23–1.39)		Reference	1.49(1.29–1.71)	
Yes	Reference	1.40(1.21–1.63)		Reference	1.50(1.05–2.14)	
Current smoking			0.393			0.362
No	Reference	1.29(1.20–1.38)		Reference	1.54(1.32–1.80)	
Yes	Reference	1.36(1.24–1.49)		Reference	1.35(1.07–1.70)	
Current drinking			0.807			0.501
No	Reference	1.33(1.24–1.42)		Reference	1.53(1.31–1.79)	
Yes	Reference	1.32(1.20–1.45)		Reference	1.40(1.11–1.78)	
Physical activity			0.705			0.729
Low	Reference	1.37(1.16–1.61)		Reference	1.23(0.76–1.99)	
Moderate	Reference	1.30(1.22–1.39)		Reference	1.48(1.27–1.73)	
High	Reference	1.36(1.21–1.53)		Reference	1.61(1.21–2.13)	
Sleep duration (h/d)			0.032			0.184
7–8	Reference	1.44(1.31–1.58)		Reference	1.68(1.34–2.09)	
<7 or >8	Reference	1.27(1.18–1.36)		Reference	1.40(1.19–1.64)	

Fig. S4 Stratified analyses for the associations between metabolic syndrome (MetS) and risk of sepsis and 28-day mortality following sepsis in the Kailuan Study (*n*=152,317). Multivariable models were adjusted for age, sex, educational level, assessment center, smoking status, alcohol consumption, physical activity, and sleep duration. CI. Confidence interval; *HR*. Hazard ratio

Table S1 Variables and medication codes used to define metabolic syndrome (MetS) in the UK Biobank

MetS component	Harmonized criteria (2009) 3 or more of the following	UK Biobank data field ID	Field code description
Elevated waist circumference	≥102 cm in males and ≥88 cm in females	48	Waist circumference
Hypertriglyceridemia	≥150 mg/dl (1.7 mmol/L)	30870	Triglycerides blood biochemistry
Elevated blood pressure	Systolic blood pressure (BP): ≥130 mmHg and/or diastolic BP: ≥85 mmHg*, or anti-hypertensive drug treatment in a patient with a history of hypertension<br>The history of hypertension was assessed based on hospital inpatient records, “first occurrences”, and self-reported medical conditions	4080, 4079, 93, 94, 20003	Systolic BP - automated reading, Diastolic BP - automated reading, Systolic BP - manual reading, Diastolic BP - manual reading, ATC codes starting with: C02, C03, C07, C08, C09
Hyperglycemia	≥ 100 mg/dl (≥ 5.6 mmol/L)†, or drug treatment for elevated glucose level	30750, 20003	HbA1c, ATC codes starting with: A10
Reduced HDL-cholesterol	Males: <40 mg/dl (1.0 mmol/L); Females: <50 mg/dl (1.3 mmol/L), or use of lipid-modifying medications	30760, 20003	HDL cholesterol, ATC codes starting with: C10

*In the UK Biobank, BP was measured twice by a trained nurse after participants had been at rest for at least 5 min. Individual systolic and diastolic BP measurements were averaged within a visit. Automated BP readings were the preferred source of data. However, if this was unavailable, manual readings were used. †HbA1c was used as a proxy indicator, with cut-offs based on the recommendations of the American Diabetes Association: HbA1c ≥5.7%. ATC. Anatomical Therapeutic Chemical; HbA1c. Glycated haemoglobin A1c; HDL. High-density lipoprotein

Table S2 Detailed description of medication codes (mapped to ATC codes) used to define metabolic syndrome (MetS) components in the UK Biobank

UKB drug category	UKB drug name	UKB code	ATC code
Medications for reduced HDL-cholesterol			
Atorvastatin	Atorvastatin	1141146234	C10AA05
Lipitor 10 mg tablet	Atorvastatin	1141146138	C10AA05
Rosuvastatin	Rosuvastatin	1141192410	C10AA07
Crestor 10 mg tablet	Rosuvastatin	1141192414	C10AA07
Simvastatin	Simvastatin	1140861958	C10AA01
Simvador 10 mg tablet	Simvastatin	1141188146	C10AA01
Zocor 10 mg tablet	Simvastatin	1140881748	C10AA01
Pravastatin	Pravastatin	1140888648	C10AA03
Lipostat 10 mg tablet	Pravastatin	1140861970	C10AA03
Ezetimibe	Ezetimibe	1141192736	C10AX09
Ezetrol 10 mg tablet	Ezetimibe	1141192740	C10AX09
Fenofibrate	Fenofibrate	1140861954	C10AB05
Lipantil micro 67 mg capsule	Fenofibrate	1141162544	C10AB05
Supralip 160 mg modified release (m/r) tablet	Fenofibrate	1141172214	C10AB05
Fluvastatin	Fluvastatin	1140888594	C10AA04
Lescol 20 mg capsule	Fluvastatin	1140864592	C10AA04
Gemfibrozil	Gemfibrozil	1140861856	C10AB04
Omacor 1 g capsule	Omega-3-acid ethyl esters	1141181868	C10AX06
Bezafibrate	Bezafibrate	1140861924	C10AB02
Questran 4 g/sachet powder	Cholestyramine	1140861936	C10AC01
Colestyramine	Cholestyramine	1140909780	C10AC01

UKB drug category	UKB drug name	UKB code	ATC code
Ciprofibrate	Ciprofibrate	1140862026	C10AB08
Bezalip-mono 400 mg m/r tablet	Bezafibrate	1140861928	C10AB02
Bezalip 200 mg tablet	Bezafibrate	1140861926	C10AB02
Niacin	Niacin	1140910670	C04AC01/C10AD02
Niaspan 500 mg m/r tablet	Niacin	1141188546	C10AD02
Nicotinic acid product	Niacin	1140861868	C04AC01/C10AD02
Cholestyramine	Cholestyramine	1140865576	C10AC01
Fibrazate extended release (or prolonged release) (xl) 400 mg m/r tablet	Bezafibrate	1141201306	C10AB02
Colestyramine product	Cholestyramine	1141180734	C10AC01
Bezafibrate product	Bezafibrate	1141157260	C10AB02
Modalim 100 mg tablet	Ciprofibrate	1140862028	C10AB08
Zimbacol xl 400 mg m/r tablet	Bezafibrate	1141171548	C10AB02
Colestipol	Colestipol	1140888590	C10AC02
Colestid 5 g/sachet granules	Colestipol	1140861848	C10AC02
Acipimox	Acipimox	1140861892	C10AD06
Colestyramine+aspartame 4 g/sachet powder	Cholestyramine	1141180722	C10AC01
Medications for elevated blood pressure			
Hydralazine	Hydralazine	1140888686	C02DB02
Moxonidine	Moxonidine	1140928284	C02AC05
Physiotens 200 µg tablet	Moxonidine	1140928290	C02AC05
Minoxidil	Minoxidil	1140860532	C02DC01/D11AX01
Doxazosin	Doxazosin	1140879778	C02CA04
Methyldopa	Methyldopa	1140860470	C02AB

UKB drug category	UKB drug name	UKB code	ATC code
Cardura 1 mg tablet	Doxazosin	1140860690	C02CA04
Doxadura 1 mg tablet	Doxazosin	1141194372	C02CA04
Clonidine	Clonidine	1140883468	C02AC01/N02CX02/S01EA04
Indoramin	Indoramin	1140879782	C02CA02
Prazosin	Prazosin	1140879794	C02CA01
Hypovase 500 mcg tablet	Prazosin	1140860580	C02CA01
Indapamide	Indapamide	1140866078	C03BA11
Natrilix sustained release (sr) 1.5 mg m/r tablet	Indapamide	1141146378	C03BA11
Hydrochlorothiazide	Hydrochlorothiazide	1140866162	C03AA03
Furosemide	Furosemide	1140909708	C03CA01
Frusemide	Furosemide	1140866116	C03CA01
Spironolactone	Spironolactone	1140866236	C03DA01
Spirolone 25 mg tablet	Spironolactone	1140866318	C03DA01
Chlortalidone	Chlorthalidone	1140909706	C03BA04
Eplerenone	Eplerenone	1141201244	C03DA04
Hygroton 50 mg tablet	Chlorthalidone	1140866146	C03BA04
Metolazone	Metolazone	1140866092	C03BA08
Bendroflumethiazide	Bendroflumethiazide	1141194794	C03AA01
Bendrofluazide	Bendroflumethiazide	1140866122	C03AA01
Co-amilofruse	Amiloride/Furosemide	1140923402	C03EB01
Bumetanide	Bumetanide	1140866280	C03CA02
Co-amilozide	Amiloride/Hydrochlorothiazide	1140923276	C03EA01

UKB drug category	UKB drug name	UKB code	ATC code
Amiloride	Amiloride	1140888512	C03DB01
Bendroflumethiazide+potassium 2.5 mg/7.7 mmol m/r tablet	Bendroflumethiazide/Potassium	1141194800	C03AB01
Frumil tablet	Amiloride/Furosemide	1140866406	C03CA01
Bendrofluazide+potassium 2.5 mg/7.7 mmol m/r tablet	Bendroflumethiazide/Potassium	1140866450	C03AB01
Bzt - bendrofluazide	Bendroflumethiazide	1140910442	C03AA01
Dyazide tablet	Triamterene/Hydrochlorothiazide	1140866402	C03EA01
Moduretic tablet	Amiloride/Hydrochlorothiazide	1140866420	C03EA01
Navispare tablet	Amiloride/Cyclopenthiazide	1140866352	C03EA07
Co-triamterzide	Triamterene/Hydrochlorothiazide	1140923272	C03EA01
Torasemide	Torasemide	1140888496	C03CA04
Moduret 25 tablet	Amiloride/Hydrochlorothiazide	1140866416	C03EA01
Burinex a tablet	Bumetanide	1140866356	C03CA02
Xipamide	Xipamide	1140866108	C03BA10
Cyclopenthiazide	Cyclopenthiazide	1140866156	C03AA07
Bisoprolol	Bisoprolol	1140879760	C07AB07
Cardicor 1.25 mg tablet	Bisoprolol	1141171152	C07AB07
Atenolol	Atenolol	1140866738	C07AB03
Aenormin 25 tablet	Atenolol	1140866756	C07AB03
Metoprolol	Metoprolol	1140879818	C07AB02
Carvedilol	Carvedilol	1140909368	C07AG02
Propranolol	Propranolol	1140879842	C07AA05
Timolol	Timolol	1140879866	C07AA06/S01ED01
Half-inderal la 80 mg m/r capsule	Propranolol	1140866800	C07AA05

UKB drug category	UKB drug name	UKB code	ATC code
Inderal 10 mg tablet	Propranolol	1140866804	C07AA05
Bedranol 10 mg tablet	Propranolol	1140851556	C07AA05
Half beta-prograne 80 mg m/r capsule	Propranolol	1140866802	C07AA05
Labetalol	Labetalol	1140879824	C07AG01
Sotalol	Sotalol	1140879854	C07AA07
Beta-cardone 40 mg tablet	Sotalol	1140860304	C07AA07
Co-tenidone	Chlorthalidone/Atenolol	1140923336	C07CB03
Nebivolol	Nebivolol	1141164276	C07AB12
Bisoprolol fumarate+hydrochlorothiazide 10 mg/6.25 mg tablet	Bisoprolol/Hydrochlorothiazide	1140864950	C07BB07
Celiprolol	Celiprolol	1140879762	C07AB08
Atenolol+bendroflumethiazide	Atenolol/Bendroflumethiazide	1141194810	C07BB03
Nebilet 5mg tablet	Nebivolol	1141164280	C07AB12
Propranolol hydrochloride+bendrofluazide 80 mg/2.5 mg capsule	Bendroflumethiazide/Propranolol	1140860418	C07BA05
Sotalol hydrochloride+hydrochlorothiazide 80 mg/12.5 mg tablet	Sotalol/Hydrochlorothiazide	1140860332	C07BA07
Tenoret 50 tablet	Chlorthalidone/Atenolol	1140860324	C07CB03
Carteolol	Carteolol	1140879822	C07AA15/S01ED05
Betaxolol	Betaxolol	1140879758	C07AB05/S01ED02
Atenolol+bendrofluazide	Atenolol/Bendroflumethiazide	1141146126	C07BB03
Tenif capsule	Atenolol/Nifedipine	1140860358	C07FB03
Metoprolol tartrate+chlorthalidone 100 mg/12.5 mg tablet	Metoprolol/Chlorthalidone	1140860308	C07CB02
Tenoretic tablet	Chlorthalidone/Atenolol	1140860328	C07CB03
Beta-adalat capsule	Atenolol/Nifedipine	1140860356	C07FB03
Celectol 200 mg tablet	Celiprolol	1140860498	C07AB08

UKB drug category	UKB drug name	UKB code	ATC code
Acebutolol	Acebutolol	1140866724	C07AB04
Oxprenolol	Oxprenolol	1140879830	C07AA02
Prindolol	Pindolol	1140910614	C07AA17
Kalten capsule	Atenolol/Amiloride/Hydrochlorothiazide	1140860398	C07DB01
Nadolol	Nadolol	1140860192	C07AA12
Pindolol	Pindolol	1140860292	C07AA03
Atenolol+chlortalidone	Chlorthalidone/Atenolol	1141180778	C07BB03
Amlodipine	Amlodipine	1140879802	C08CA01
Istin 5 mg tablet	Amlodipine	1140861202	C08CA01
Amlostin 5 mg tablet	Amlodipine	1141200400	C08CA01
Nifedipine	Nifedipine	1140861088	C08CA05
Adalat 5 mg capsule	Nifedipine	1140861090	C08CA05
Coracten sr 10 mg m/r capsule	Nifedipine	1140861120	C08CA05
Adalate 10 mg capsule	Nifedipine	1140881702	C08CA05
Adipine mr 10 m/r tablet	Nifedipine	1140923572	C08CA05
Fortipine la40 m/r tablet	Nifedipine	1141145870	C08CA05
Nifedipress mr 10 m/r tablet	Nifedipine	1141157140	C08CA05
Tensipine mr 10 m/r tablet	Nifedipine	1140927940	C08CA05
Verapamil	Verapamil	1140888510	C08DA01
Securon 40 mg tablet	Verapamil	1140866466	C08DA01
Half securon sr 120 mg m/r tablet	Verapamil	1140866460	C08DA01
Univer 120 mg m/r capsule	Verapamil	1140881692	C08DA01
Vertab sr 240 m/r tablet	Verapamil	1141169710	C08DA01

UKB drug category	UKB drug name	UKB code	ATC code
Diltiazem	Diltiazem	1140879806	C05AE03/C08DB01
Tildiem 60 mg m/r tablet	Diltiazem	1140861128	C08DB01
Adizem-60 m/r tablet	Diltiazem	1140861138	C08DB01
Adizem-xl plus m/r capsule	Diltiazem	1140926780	C08DB01
Dilzem sr 60 mg long acting m/r capsule	Diltiazem	1140861166	C08DB01
Slozem 120 mg m/r capsule	Diltiazem	1140911698	C08DB01
Angitil sr 90 m/r capsule	Diltiazem	1140917428	C08DB01
Viazem xl 120 mg m/r capsule	Diltiazem	1141151474	C08DB01
Zemtard 120 xl m/r capsule	Diltiazem	1141167832	C08DB01
Calcicard 60 mg tablet	Diltiazem	1140851730	C08DB01
Felodipine	Felodipine	1140888646	C08CA02
Cardioplen xl 5 mg m/r tablet	Felodipine	1141199858	C08CA02
Vascalpha 5 mg m/r tablet	Felodipine	1141190160	C08CA02
Felendil xl 5 mg m/r tablet	Felodipine	1141188836	C08CA02
Plendil 2.5 mg m/r tablet	Felodipine	1140928212	C08CA02
Felotens xl 5 mg m/r tablet	Felodipine	1141188152	C08CA02
Neofel xl 5 mg m/r tablet	Felodipine	1141200782	C08CA02
Felogen xl 5 mg m/r tablet	Felodipine	1141188576	C08CA02
Cabren 2.5 mg m/r tablet	Felodipine	1141187094	C08CA02
Lercanidipine	Lercanidipine	1141153026	C08CA13
Zanidip 10 mg tablet	Lercanidipine	1141153032	C08CA13
Lacidipine	Lacidipine	1140861276	C08CA09
Diltiazem hcl+hydrochlorothiazide 150 mg/12.5 mg m/r capsule	Diltiazem/Hydrochlorothiazide	1140926778	C08GA

UKB drug category	UKB drug name	UKB code	ATC code
Nicardipine	Nicardipine	1140879810	C08CA04
Motens 2 mg tablet	Lacidipine	1140861282	C08CA09
Cardene 20 mg capsule	Nicardipine	1140861176	C08CA04
Valsartan	Valsartan	1141145660	C09CA03
Diovan 40 mg capsule	Valsartan	1141145668	C09CA03
Losartan	Losartan	1140916356	C09CA01
Cozaar 25 mg tablet	Losartan	1141179974	C09CA01
Perindopril	Perindopril	1140888560	C09AA04
Coversyl 2 mg tablet	Perindopril	1140860802	C09AA04
Lisinopril	Lisinopril	1140860696	C09AA03
Zestril 2.5 mg tablet	Lisinopril	1140860714	C09AA03
Irbesartan	Irbesartan	1141152998	C09CA04
Zprovel 75 mg tablet	Irbesartan	1141153006	C09CA04
Enalapril	Enalapril	1140888552	C09AA02
Innovace 2.5 mg tablet	Enalapril	1140860776	C09AA02
Fosinopril	Fosinopril	1140888556	C09AA09
Ramipril	Ramipril	1140860806	C09AA05
Telmisartan	Telmisartan	1141166006	C09CA07
Micardis 20 mg tablet	Telmisartan	1141172492	C09CA07
Tritace 1.25 mg tablet	Ramipril	1141188408	C09AA05
Lopace 2.5 mg capsule	Ramipril	1141199940	C09AA05
Candesartan cilexetil	Candesartan	1141156836	C09CA06
Amias 2 mg tablet	Candesartan	1141156846	C09CA06

UKB drug category	UKB drug name	UKB code	ATC code
Cilazapril	Cilazapril	1140860882	C09AA08
Olmesartan	Olmesartan	1141193282	C09CA08
Losartan potassium+hydrochlorothiazide 50 mg/12.5 mg tablet	Losartan/Hydrochlorothiazide	1141151016	C09DA01
Olmetec 10 mg tablet	Olmesartan	1141193346	C09CA08
Trandolapril	Trandolapril	1140860904	C09AA10
Eprosartan	Eprosartan	1141171336	C09CA02
Captopril	Captopril	1140860750	C09AA01
Quinapril	Quinapril	1140860728	C09AA06
Lisinopril+hydrochlorothiazide 10 mg/12.5 mg tablet	Lisinopril/Hydrochlorothiazide	1140864952	C09BA03
Coaprovel 150 mg/12.5 mg tablet	Hydrochlorothiazide/Irbesartan	1141172686	C09DA04
Enalapril maleate+hydrochlorothiazide 20 mg/12.5 mg tablet	Enalapril/Hydrochlorothiazide	1140860790	C09BA02
Irbesartan+hydrochlorothiazide 150 mg/12.5 mg tablet	Hydrochlorothiazide/Irbesartan	1141172682	C09DA04
Cozaar-comp 50 mg/12.5 mg tablet	Losartan/Hydrochlorothiazide	1141151018	C09DA01
Perindopril+indapamide	Perindopril/Indapamide	1141180592	C09BA04
Zestoretic 10 tablet	Lisinopril/Hydrochlorothiazide	1140864618	C09BA03
Coversyl plus 4 mg/1.25 mg tablet	Perindopril/Indapamide	1141180598	C09BA04
Co-diovan 80 mg/12.5 mg tablet	Valsartan/Hydrochlorothiazide	1141201040	C09DA03
Valsartan+hydrochlorothiazide 80 mg/12.5 mg tablet	Valsartan/Hydrochlorothiazide	1141201038	C09DA03
Teveten 300 mg tablet	Eprosartan	1141171344	C09CA02
Micardisplus 40 mg/12.5 mg tablet	Telmisartan/Hydrochlorothiazide	1141187790	C09DA07
Telmisartan+hydrochlorothiazide 40 mg/12.5 mg tablet	Telmisartan/Hydrochlorothiazide	1141187788	C09DA07
Felodipine+ramipril	Ramipril/Felodipine	1141165470	C09BB05
Imidapril hydrochloride	Imidapril	1141164148	C09AA16

UKB drug category	UKB drug name	UKB code	ATC code
Innozide tablet	Enalapril/Hydrochlorothiazide	1140860784	C09BA02
Gopten 500 micrograms capsule	Trandolapril	1140860912	C09AA10
Capozide tablet	Hydrochlorothiazide/Captopril	1140881714	C09BA01
Capoten 12.5 mg tablet	Captopril	1140860758	C09AA01
Carace 10 plus tablet	Lisinopril/Hydrochlorothiazide	1140864910	C09BA03
Triapin mite 2.5 mg/2.5 mg tablet	Ramipril/Felodipine	1141165476	C09BB05
Accupro 5 mg tablet	Quinapril	1140881706	C09AA06
Medications for elevated blood glucose			
Metformin	Metformin	1140884600	A10BA02
Glucophage 500 mg tablet	Metformin	1140874686	A10BA02
Insulin product	Insulin	1140883066	A10A
Gliclazide	Gliclazide	1140874744	A10BB09
Glyclizide	Gliclazide	1140910566	A10BB09
Diamicron 80 mg tablet	Gliclazide	1140874746	A10BB09
Glimepiride	Glimepiride	1141152590	A10BB12
Amaryl 1 mg tablet	Glimepiride	1141156984	A10BB12
Pioglitazone	Pioglitazone	1141171646	A10BG03
Actos 15 mg tablet	Pioglitazone	1141171652	A10BG03
Glibenclamide	Glyburide	1140874718	A10BB01
Repaglinide	Repaglinide	1141168660	A10BX02
Rosiglitazone	Rosiglitazone	1141177600	A10BG02
Rosiglitazone 1 mg/metformin 500 mg tablet	Metformin/Rosiglitazone	1141189090	A10BD03
Avandamet 1 mg/500 mg tablet	Metformin/Rosiglitazone	1141189094	A10BD03

UKB drug category	UKB drug name	UKB code	ATC code
Glipizide	Glipizide	1140874646	A10BB07
Avandia 4mg tablet	Rosiglitazone	1141177606	A10BG02
Tolbutamide	Tolbutamide	1140874674	A10BB03/V04CA01
Acarbose	Acarbose	1140868902	A10BF01
Nateglinide	Nateglinide	1141173882	A10BX03

ATC. Anatomical Therapeutic Chemical; HDL. High-density lipoprotein; UKB. UK Biobank

Table S3 Ascertainment of sepsis and cancer in the UK Biobank

Disease	ICD-9	ICD-10	Self-reported fields
Sepsis	0031, 0223, 0270, 038, 670	A021, A227, A327, A40, A41, A427, B377, R651, R572, O85	-
Cancer	140–209	C00–C97	2453, 20001

ICD-9. The 9th revision of the International Classification of Diseases; ICD-10. The 10th revision of the International Classification of Diseases; "-" indicates that no self-reported field was used for the ascertainment of sepsis

Table S4 International Classification of Diseases (ICD) codes for identification of infection in the Kailuan Study

Code description	ICD-10
Cholera	A00
Typhoid/paratyphoid fever	A01
Other salmonella infection	A02, G01, J17, M01, M90
Shigellosis	A03
Other food poisoning	A05
Intestinal infection not otherwise classified	A02, A04, A08
Ill-defined intestinal infection	A09
Primary tuberculosis	A15, A16
Pulmonary tuberculosis	A15, A16
Other respiratory tuberculosis	A15, A16, J38
Central nervous system tuberculosis	A17
Intestinal tuberculosis	A18, K93
Tuberculosis of bone and joint	A18, H75
Genitourinary tuberculosis	A18, N51, N74
Tuberculosis not otherwise classified	A18, D77, E35, K23
Military tuberculosis	A19
Plague	A20
Tularemia	A21
Anthrax	A22
Brucellosis	A23
Glanders	A24
Melioidosis	A24
Rat-bite fever	A25
Other bacterial zoonoses	A26, A28, A32
Leprosy	A30
Other mycobacterial disease	A31
Diphtheria	A36, I41, K67, N33
Whooping cough	A37
Streptococcal throat/scarlet fever	A38, J02
Erysipelas	A46
Meningococcal infection	A39, G05, H48, M01
Tetanus	A35
Septicemia	A40, A41
Actinomycotic infections	A42, A43, B47
Other bacterial diseases	A48, K90, M60
Bacterial infection in other diseases not otherwise specified	B95, B96
Congenital syphilis	A50
Early symptomatic syphilis	A51, R59, H22, H32, M90, K77, G01, L99
Early syphilis latent	A51
Cardiovascular syphilis	A52, I32, I39, I41, I79
Neurosyphilis	A52, G01, G05, H32, H48, H94, I60,
Other late symptomatic syphilis	A52, H19, J99, K67, K77, N29,

Code description	ICD-10
	M63, M68, M90
Late syphilis latent	A52
Other and unspecified syphilis	A52, A53
Gonococcal infections	A54, N30, N33, N39, N51, N72, N74, H19, G01, I32, I39, I52, K67
Leptospirosis	A27, G01
Vincent’s angina	A69
Yaws	A66
Pinta	A67
Other spirochetal infection	A65, A69
Dermatophytosis	B35
Dermatomycosis not otherwise classified or specified	B36
Candidiasis	A09, B37, H60
Coccidioidomycosis	B38
Histoplasmosis	B39, G02, H36, I32, I39, J99
Blastomycotic infection	B40, B41, B48
Other mycoses	B42, B43, B44, B45, B46, B47, B48
Opportunistic mycoses	B48
Bacterial meningitis	G00
Meningitis, unspecified	G03, G05
Central nervous system abscess	G06
Phlebitis of intracranial sinus	G08
Acute pericarditis	I30, I32
Acute or subacute endocarditis	I33, I39
Thrombophlebitis	I80
Acute sinusitis	J01
Acute pharyngitis	J02
Acute tonsillitis	J03
Acute laryngitis/tracheitis	J04, J05
Acute upper respiratory infection of multiple sites/not otherwise specified	J06
Pneumococcal pneumonia	J13
Other bacterial pneumonia	J14, J15
Bronchopneumonia with organism not otherwise specified	J18
Pneumonia, organism not otherwise specified	J18
Acute exacerbation of obstructive chronic bronchitis	J44.1
Bronchiectasis	J47
Empyema	J86
Lung/mediastinum abscess	J85
Acute appendicitis	K35
Appendicitis not otherwise specified	K37
Other appendicitis	K36
Diverticulitis of small intestine without hemorrhage	K57.12
Diverticulitis of small intestine with hemorrhage	K57.13
Diverticulitis of colon without hemorrhage	K57.22
Diverticulitis of colon with hemorrhage	K57.23

Code description	ICD-10
Anal and rectal abscess	K61
Peritonitis	K65, K67
Intestinal abscess	K63.0
Perforation of intestine	K63.1
Abscess of liver	K75.0
Portal pyema	K75.1
Acute cholecystitis	K81.0
Kidney infection	N10, N11, N12, N15
Urethritis/urethral syndrome	N34
Urinary tract infection not otherwise specified	N39.0
Prostatic inflammation	N41, N51
Female pelvic inflammation disease	N70, N73
Uterine inflammation disease	N71
Other female genital inflammation	N72, N75, N76, N77
Cellulitis, finger/toe	L03
Other cellulitis or abscess	L03
Acute lymphadenitis	L04
Other local skin infection	L08, L98
Pyogenic arthritis	M00.9
Osteomyelitis	M86, M89, M90
Bacteremia	A49.9
Infection or inflammation of device/graft	T82.6, T82.7, T83.5, T84.5, T84.6, T85.71, T85.78, T85.81
Postoperative infection	T81.4
Infectious complication of medical care not otherwise classified	T80.2
Bacterial infection of unspecified site	A49
Typhus fever	A75
Spotted fever (tick-borne rickettsioses)	A77
Q fever	A78
Other rickettsioses	A79
Acute poliomyelitis	A80
Atypical virus infections of central nervous system	A81
Rabies	A82
Mosquito-borne viral encephalitis	A83
Tick-borne viral encephalitis	A84
Other viral encephalitis, not elsewhere classified	A85
Unspecified viral encephalitis	A86
Viral meningitis	A87
Other viral infections of central nervous system, not elsewhere classified	A88
Unspecified viral infection of central nervous system	A89
Dengue fever (classical dengue)	A90
Dengue hemorrhagic fever	A91
Other mosquito-borne viral fevers	A92
Other arthropod-borne viral fevers, not elsewhere classified	A93
Unspecified arthropod-borne viral fever	A94

Code description	ICD-10
Yellow fever	A95
Arenaviral hemorrhagic fever	A96
Other viral hemorrhagic fevers, not elsewhere classified	A98
Unspecified viral hemorrhagic fever	A99
Other viral diseases, not elsewhere classified	B33
Viral infection of unspecified site	B34
Unspecified mycosis	B49
Plasmodium falciparum malaria	B50
Plasmodium vivax malaria	B51
Plasmodium malariae malaria	B52
Other specified malaria	B53
Unspecified malaria	B54
Leishmaniasis	B55
African trypanosomiasis	B56
Chagas’ disease	B57
Toxoplasmosis	B58
Pneumocystosis	B59
Other protozoal diseases, not elsewhere classified	B60
Unspecified protozoal disease	B64
Schistosomiasis (bilharziasis)	B65
Other fluke infections	B66
Echinococcosis	B67
Taeniasis	B68
Cysticercosis	B69
Diphyllobothriasis and sparganosis	B70
Other cestode infections	B71
Dracunculiasis	B72
Onchocerciasis	B73
Filariasis	B74
Trichinellosis	B75
Hookworm diseases	B76
Ascariasis	B77
Strongyloidiasis	B78
Trichuriasis	B79
Enterobiasis	B80
Other intestinal helminthiases, not elsewhere classified	B81
Unspecified intestinal parasitism	B82
Other helminthiases	B83
Pediculosis and phthiriasis	B85
Scabies	B86
Myiasis	B87
Other infestations	B88
Unspecified parasitic disease	B89
Sequelae of tuberculosis	B90

Table S5 International Classification of Diseases (ICD) codes for identification of organ dysfunction in the Kailuan Study

System	ICD-10	Codes description
Cardiovascular	A41.9	Septic shock
	A48.3	Toxic shock syndrome
	E86.x00	Volume depletion
	E86.x00x001	Hypovolemia
	E86.x00x003	Extracellular fluid deletion
	E86.x00x004	Plasma volume depletion
	E86.x00x005	Volume depletion
	E86.x01	Dehydration
	I51.400x007	Severe Myocarditis
	I95.8	Hypotension, unspecified
	I95.9	Hypotension
	I99.x00	Other disorders of the circulatory system
	I99.x01	Circulatory disorders
	R09.800x082	Weak pulse
	R57.0	Cardiogenic shock
	R57.1	Hypovolaemic shock
	R57.2	Septic shock
	R57.8	Other shock
	R57.9	Shock, unspecified
	O03.300x001	Spontaneous abortion, incomplete, with septic shock
	O03.800x001	Spontaneous abortion, complete, with septic shock
	O04.300x004	Medical abortion, incomplete, with septic shock
	O04.800x001	Medical abortion, complete, with septic shock
	O04.804	Medical abortion, later complete, with septic shock
	P29	Cardiovascular disorders originating in the perinatal period
Respiratory	J80	Acute respiratory distress syndrome
	J81	Pulmonary oedema
	J95.100	Acute pulmonary insufficiency after thoracic surgery
	J95.200	Acute pulmonary insufficiency after non-thoracic surgery
	J95.800x004	Respiratory failure after surgical procedures
	J95.800x021	Respiratory distress syndrome in adults after surgery
	J96	Acute respiratory failure with hypoxia, not elsewhere classified
	J96.9	Respiratory failure, unspecified with hypoxia
	J98.4	Other disorders of lung
	J98.400x024	Severe infection of the lungs
	P22	Respiratory distress of newborn
	P28.5	Respiratory failure of newborn
	R09.0	Asphyxia
	R09.000	Suffocate
	R09.2	Respiratory arrest
	R09.800x095	Asthma

System	ICD-10	Codes description
	U04.9	Severe acute respiratory syndrome (SARS), unspecified
Central nervous system	F05	Delirium (not alcohol- or drug-induced)
	F06.8	Other specified mental disorders due to infection
	G93.1	Anoxic brain damage, not elsewhere classified
	G93.4	Encephalopathy, unspecified
	G93.8	Other specified disorders of brain
	G93.9	Disorder of brain, unspecified
	R40	Somnolence, stupor and coma
	R41.0	Disorientation, unspecified
	R45.3	Demoralization and apathy
	R55	Syncope and collapse
Renal	A98.500	Haemorrhagic fever with renal syndrome
	N17	Acute kidney failure
	N19	Unspecified kidney failure
	R34	Anuria and oliguria
	R39.2	Extrarenal uraemia
	R94.4	Abnormal results of kidney function studies
Metabolic	E87.2	Acidosis
Hematologic	A93.800x001	Other specified arthropod-borne viral fevers (Piry virus disease)
	D61.900x001	Bone marrow suppression
	D61.901	Myelosuppressive anemia
	D61.903	Pancytopenia
	D61.906	Acute bone marrow hematopoietic function inhibition
	D65	Disseminated intravascular coagulation (defibrination syndrome)
	D65.x00x003	Gangrene purpura
	D65.x01	Acquired fibrinogen deficiency
	D65.x02	Acquired fibrinolytic bleeding
	D65.x03	Fibrinolytic purpura
	D68.9	Coagulation defect, unspecified
	D69.000x008	Infectious purpura
	D69.000x011	Bacterial purpura
	D69.000x013	Toxic purpura
	D69.203	Purpura
	D69.301	Hemorrhagic purpura
	D69.5	Secondary thrombocytopenia
	D69.501	Secondary thrombocytopenic purpura
	D69.6	Thrombocytopenia, unspecified
	D69.8	Other specified haemorrhagic conditions
	D76.200x001	Infectious hemophagocytic syndrome
	D76.200x011	Infectious erythrophagocytic syndrome
Hepatic	B15.000	Hepatitis A, accompanied by hepatic coma

System	ICD-10	Codes description
	B15.001	Acute viral hepatitis A with hepatic coma
	B15.002	Acute severe severe viral hepatitis A with hepatic coma
	B15.003	Subacute severe viral hepatitis A with hepatic coma
	B16.000	Acute hepatitis B, with δ factor (co-infection) and accompanied by hepatic coma
	B16.001	Acute hepatitis B–D with hepatic coma
	B16.200	Acute hepatitis B, not accompanied by δ factor (co-infection), but with hepatic coma
	B16.201	Acute viral hepatitis B with hepatic coma
	B16.202	Subacute severe viral hepatitis B with hepatic coma
	B16.203	Acute severe hepatitis B with hepatic coma
	B16.204	Acute jaundice-free hepatitis B with hepatic coma
	B16.206	Acute severe hepatitis B with hepatic coma
	B17.807	Acute severe hepatitis hepatitis
	B19.000	Viral hepatitis, accompanied by hepatic coma
	B19.000x001	Viral hepatitis with hepatic coma
	B19.001	Acute severe viral hepatitis with hepatic coma
	B19.002	Subacute severe viral hepatitis with hepatic coma
	B25.101†	Cytome hepatitis with hepatic coma
	E80.600	Bilirubin metabolism disorders, others
	E80.604	Hyperbilirubinemia
	E80.700	Bilirubin metabolism disorders
	K71.100x001	Toxic liver disease with liver failure
	K71.103	Toxic liver failure
	K72.0	Acute and subacute hepatic failure
	K72.9	Hepatic failure, unspecified
	K76.7	Hepatorenal syndrome
	K76.8	Other specified diseases of liver
	K76.9	Liver disease, unspecified
	K91.825	Liver failure after surgery
Others	A01.003	Typhoid fever sepsis
	A02.100	Salmonella sepsis
	A03.900	Shigellosis, unspecified (shock)
	A09.005	Septic gastroenteritis
	A20.7	Septicemic plague
	A21.7	Generalized tularemia
	A22.7	Anthrax sepsis
	A24.1	Acute and fulminating melioidosis
	A26.7	Erysipelothrix sepsis
	A27.900	Leptospirosis
	A28.001	Pasteurellosis
	A28.2	Extraintestinal yersiniosis
	A32.7	Listerial sepsis
	A38.x00x012	Scarlet fever sepsis

System	ICD-10	Codes description
	A39.2	Acute meningococcemia
	A39.3	Chronic meningococcemia
	A39.4	Meningococcemia, unspecified
	A39.1	Waterhouse
	A40.	Streptococcal sepsis
	A41.	Other sepsis
	A42.7	Actinomycotic sepsis
	A49.103	Streptococcal infection syndrome
	A54.8	Other gonococcal infections (sepsis)
	A88.800x001	Polio-like syndrome
	B00.7	Herpetic septicemia
	B37.7	Candidal sepsis
	B37.6	Candidal endocarditis
	B49	Unspecified mycosis (Fungemia)
	D71.x00x005	Progressive septic granulomatous disease
	J15.903	Bacterial pneumonia, unspecified (severe community-acquired)
	J18.903	Pneumonia, unspecified (severe)
	J95.000x001	Sepsis of tracheostomy stoma
	O08.000x006	Sepsis following abortion and ectopic and molar pregnancy
	O08.200x002	Embolism following abortion and ectopic and molar pregnancy (septic)
	O08.200x006	Embolism following abortion and ectopic and molar pregnancy (septicopyaemic)
	O85	Puerperal sepsis
	O88.300	Obstetric pyaemic and septic embolism
	P36	Bacterial sepsis of newborn
	P37.800x002	Other specified congenital infectious and parasitic diseases
	R09.800	Involves other specific signs and symptoms of the circulatory and respiratory systems
	R65.1	Systemic inflammatory response syndrome (SIRS) due to infection with organ dysfunction
	T80.2	Infections following infusion, transfusion and therapeutic injection
	T81.4	Infection following a procedure, not elsewhere classified
	T88.000x002	Sepsis following immunization
Multiple	R68.800x001	Multiple organ failure

Table S6 Dietary components and their corresponding intake goals used to calculate the dietary score[a]

Dietary component	Intake goal	Score
Fruits	≥3 servings/d	1
Vegetables	≥3 servings/d	1
Whole grains	≥3 servings/d	1
(Shell) fish	≥2 servings/week	1
Dairy	≥2 servings/d	1
Vegetable oils	≥2 servings/d	1
Refined grains	≤2 servings/d	1
Processed meats	≤1 serving/week	1
Unprocessed meats	≤2 servings/week	1
Sugar-sweetened beverages	No consumption	1

[a]The dietary score was calculated by summing up the scores of all dietary components. A healthy diet was defined by a dietary score of 5 or above

Table S7 Number and percentage of study participants with missing data on covariates in the UK Biobank

Covariate	Number	Percentage (%)
Ethnicity	1682	0.47
Educational level	4228	1.18
Townsend deprivation index	449	0.12
Smoking status	1766	0.49
Alcohol consumption	884	0.25
Healthy diet	6880	1.91
Regular physical activity	24,394	6.78
Sleep duration	2610	0.73
Aspirin use	4660	1.30
Non-aspirin non-steroidal anti-inflammatory drug use	4660	1.30
Vitamin supplementation	2207	0.61
Mineral and other dietary supplementation	1419	0.39

Table S8 Number and percentage of study participants with missing data on covariates in the Kailuan Study

Covariate	Number	Percentage (%)
Educational level	12,618	8.3
Smoking status	6003	3.9
Alcohol consumption	5305	3.5
Physical activity	7264	4.8
Sleep duration	10,775	7.1

Table S9 Ascertainment of sepsis outcomes in the Kailuan Study by Sepsis-3 Criteria and ICD codes

Ascertainment method	No. of cases	
	**Sepsis incidence**	**28-day mortality following sepsis**
Sepsis-3 criteria[a]	4619	696
ICD codes[b]	1053	292
Total	5672	988

[a]Sepsis was identified according to the Sepsis-3 definition as suspected or documented infection accompanied by an acute increase in SOFA score of ≥2 points. [b]Sepsis was identified using ICD codes from hospital discharge diagnoses, including both explicit sepsis codes and implicit sepsis definitions (i.e., infection codes combined with organ dysfunction codes), for cases without sufficient data to apply Sepsis-3 criteria. ICD. International Classification of Diseases; Sepsis-3. Third International Consensus Definitions for Sepsis and Septic Shock; SOFA. Sequential Organ Failure Assessment

Table S10 Association between metabolic syndrome (MetS) and risk of mortality at different times following sepsis in the UK Biobank (*n*=359,633)

**Outcome**	**No MetS**	**MetS**	***P*-value**
7-day mortality following sepsis			
Cases/person-years	517/3,263,639	623/1,541,434	
Model 1[a] [*HR* (95% CI)]	Reference	1.90 (1.69–2.14)	<0.001
Model 2[b] [*HR* (95% CI)]	Reference	1.64 (1.45–1.85)	<0.001
Model 3[c] [*HR* (95% CI)]	Reference	1.52 (1.34–1.72)	<0.001
60-day mortality following sepsis			
Cases/person-years	1239/3,263,639	1429/1,541,434	
Model 1[a] [*HR* (95% CI)]	Reference	1.83 (1.69–1.97)	<0.001
Model 2[b] [*HR* (95% CI)]	Reference	1.60 (1.48–1.74)	<0.001
Model 3[c] [*HR* (95% CI)]	Reference	1.51 (1.40–1.64)	<0.001
90-day mortality following sepsis			
Cases/person-years	1384/3,263,639	1608/1,541,434	
Model 1[a] [*HR* (95% CI)]	Reference	1.84 (1.71–1.98)	<0.001
Model 2[b] [*HR* (95% CI)]	Reference	1.63 (1.51–1.75)	<0.001
Model 3[c] [*HR* (95% CI)]	Reference	1.54 (1.42–1.66)	<0.001
180-day mortality following sepsis			
Cases/person-years	1668/3,263,639	1870/1,541,434	
Model 1[a] [*HR* (95% CI)]	Reference	1.78 (1.67–1.90)	<0.001
Model 2[b] [*HR* (95% CI)]	Reference	1.58 (1.47–1.69)	<0.001
Model 3[c] [*HR* (95% CI)]	Reference	1.49 (1.39–1.60)	<0.001
1-year mortality following sepsis			
Cases/person-years	1958/3,263,639	2171/1,541,434	
Model 1[a] [*HR* (95% CI)]	Reference	1.77 (1.67–1.89)	<0.001
Model 2[b] [*HR* (95% CI)]	Reference	1.57 (1.47–1.67)	<0.001
Model 3[c] [*HR* (95% CI)]	Reference	1.49 (1.40–1.59)	<0.001

[a]Model 1: adjusted for age and sex. [b]Model 2: further adjusted for ethnicity, educational level, assessment center, Townsend deprivation index, smoking status, alcohol consumption, healthy diet, regular physical activity, and sleep duration. [c]Model 3: further adjusted for aspirin use, non-aspirin non-steroidal anti-inflammatory drug use, vitamin supplementation, and mineral and other dietary supplementation. CI. Confidence interval; *HR*. Hazard ratio

Table S11 Baseline characteristics of the study participants according to metabolic syndrome (MetS) evolution in the Kailuan Study[a]

**Characteristics**	**Overall**	**MetS evolution group**			
		**Sustained metabolic health**	**MetS recovery**	**MetS progression**	**Persistent MetS**
No. of participants	62,578	31,827	6924	11,057	12,770
Age [year, mean±SD]	53.6±12.4	51.6±12.4	56.5±11.7	53.6±11.5	56.7±10.7
Male [*n* (%)]	48,661 (77.8)	23,875 (75.0)	5662 (81.8)	8971 (81.1)	10,153 (79.5)
High school or above [*n* (%)]	16,235 (25.9)	9486 (29.8)	1380 (19.9)	2715 (24.6)	2654 (20.8)
Current smoker [*n* (%)]	20,738 (33.1)	10,288 (32.3)	2101 (30.3)	4059 (36.7)	4290 (33.6)
Current drinker [*n* (%)]	21,009 (33.6)	10,240 (32.2)	2115 (30.5)	4232 (38.2)	4422 (34.6)
Physical activity [*n* (%)]					
Low	19,464 (31.1)	10,022 (31.5)	1950 (28.2)	3598 (32.5)	3894 (30.5)
Moderate	34,082 (54.5)	17,475 (54.9)	3926 (56.7)	5825 (52.7)	6856 (53.7)
High	9032 (14.4)	4330 (13.6)	1048 (15.1)	1634 (14.8)	2020 (15.8)
Sleep duration [*n* (%)]					
Normal (7–8 h/d)	41,891 (66.9)	21,439 (67.4)	4776 (69.0)	7238 (65.5)	8438 (66.1)
Short (<7 h/d)	19,719 (31.5)	9889 (31.1)	2066 (29.8)	3648 (33.0)	4116 (32.2)
Long (>8 h/d)	968 (1.5)	499 (1.6)	82 (1.2)	171 (1.5)	216 (1.7)
Component of MetS [*n* (%)]					
Elevated waist circumference	44,020 (70.3)	16,660 (52.3)	4744 (68.5)	10,414 (94.2)	12,202 (95.6)
Hypertriglyceridemia	19,243 (30.8)	3271 (10.3)	974 (14.1)	6422 (58.1)	8576 (67.2)
Elevated blood pressure	38,920 (62.2)	13,448 (42.3)	4212 (60.8)	9591 (86.7)	11,669 (91.4)
Hyperglycemia	23,580 (37.7)	5355 (16.8)	1546 (22.3)	7310 (66.1)	9369 (73.4)
Reduced HDL-cholesterol	7127 (11.4)	1599 (5.0)	233 (3.4)	2363 (21.4)	2932 (23.0)

[a]Continuous variables are presented as mean±SD, and categorical variables are presented in number and percentage. HDL. High-density lipoprotein; SD. Standard deviation

Table S12 Associations between metabolic syndrome (MetS) and risk of sepsis incidence in the sensitivity analyses

**Sensitivity analysis**	**No MetS**	**MetS [*HR* (95% CI)]**	***P*-value**
UK Biobank[a]			
Excluding the first two years of follow-up	Reference	1.56 (1.49–1.62)	<0.001
Excluding participants with missing data on covariates	Reference	1.52 (1.45–1.58)	<0.001
Excluding participants with a fasting time of <3 h	Reference	1.57 (1.50–1.64)	<0.001
Using multiple imputation for missing data	Reference	1.54 (1.48–1.60)	<0.001
Using an alternative ascertainment method to define sepsis incidence	Reference	1.54 (1.48–1.61)	<0.001
Using non-sepsis death as a competing event	Reference	1.55 (1.49–1.61)	<0.001
Using the 2005 International Diabetes Federation criteria to define MetS	Reference	1.46 (1.41–1.52)	<0.001
Additional adjustment for a history of CVD, CKD, CLD, and COPD	Reference	1.50 (1.45–1.57)	<0.001
Kailuan Study[b]			
Excluding the first two years of follow-up	Reference	1.34 (1.26–1.41)	<0.001
Excluding participants with missing data on covariates	Reference	1.32 (1.25–1.40)	<0.001
Using multiple imputation for missing data	Reference	1.33 (1.26–1.40)	<0.001
Using non-sepsis death as a competing event	Reference	1.32 (1.25–1.40)	<0.001
Using the 2005 International Diabetes Federation criteria to define MetS	Reference	1.34 (1.27–1.42)	<0.001

[a]Multivariable model was adjusted for age, sex, ethnicity, educational level, assessment center, Townsend deprivation index, smoking status, alcohol consumption, healthy diet, regular physical activity, sleep duration, aspirin use, non-aspirin non-steroidal anti-inflammatory drug use, vitamin supplementation, and mineral and other dietary supplementation. [b]Multivariable model was adjusted for age, sex, educational level, assessment center, smoking status, alcohol consumption, physical activity, and sleep duration. CI. Confidence interval; CKD. Chronic kidney disease; CLD. Chronic liver disease; COPD. Chronic obstructive pulmonary disease; CVD. Cardiovascular disease; *HR*. Hazard ratio

Table S13 Associations between metabolic syndrome (MetS) and risk of 28-day mortality following sepsis in the sensitivity analyses

**Sensitivity analysis**	**No MetS**	**MetS [*HR* (95% CI)]**	***P*-value**
UK Biobank[a]			
Excluding the first two years of follow-up	Reference	1.50 (1.37–1.65)	<0.001
Excluding participants with missing data on covariates	Reference	1.46 (1.32–1.61)	<0.001
Excluding participants with a fasting time of <3 h	Reference	1.57 (1.41–1.73)	<0.001
Using multiple imputation for missing data	Reference	1.49 (1.36–1.63)	<0.001
Using an alternative ascertainment method to define mortality following sepsis	Reference	1.48 (1.37–1.60)	<0.001
Using non-sepsis death as a competing event	Reference	1.51 (1.37–1.65)	<0.001
Using the 2005 International Diabetes Federation criteria to define MetS	Reference	1.41 (1.29–1.55)	<0.001
Additional adjustment for a history of CVD, CKD, CLD, and COPD	Reference	1.44 (1.32–1.58)	<0.001
Kailuan Study[b]			
Excluding the first two years of follow-up	Reference	1.49 (1.31–1.69)	<0.001
Excluding participants with missing data on covariates	Reference	1.50 (1.31–1.70)	<0.001
Using multiple imputation for missing data	Reference	1.50 (1.32–1.70)	<0.001
Using non-sepsis death as a competing event	Reference	1.49 (1.32–1.69)	<0.001
Using the 2005 International Diabetes Federation criteria to define MetS	Reference	1.47 (1.29–1.68)	<0.001

[a]Multivariable model was adjusted for age, sex, ethnicity, educational level, assessment center, Townsend deprivation index, smoking status, alcohol consumption, healthy diet, regular physical activity, sleep duration, aspirin use, non-aspirin non-steroidal anti-inflammatory drug use, vitamin supplementation, and mineral and other dietary supplementation. [b]Multivariable model was adjusted for age, sex, educational level, assessment center, smoking status, alcohol consumption, physical activity, and sleep duration. CKD. Chronic kidney disease; CLD. Chronic liver disease; COPD. Chronic obstructive pulmonary disease; CVD. Cardiovascular disease

Table S14 Potential joint effect of metabolic syndrome (MetS) and lifestyle (after excluding BMI) on risk of sepsis in the UK Biobank[a,b]

Groups	Sepsis incidence		28-day mortality following sepsis	
	***HR* (95% CI)**	***P*-value**	***HR* (95% CI)**	***P*-value**
Individuals free of MetS	Reference		Reference	
Individuals with MetS				
Favorable lifestyle	1.26 (1.17–1.36)	<0.001	1.09 (0.91–1.31)	0.334
Intermediate lifestyle	1.50 (1.41–1.59)	<0.001	1.46 (1.27–1.67)	<0.001
Unfavorable lifestyle	1.87 (1.77–1.96)	<0.001	1.85 (1.65–2.07)	<0.001

[a]Adjusted for age, sex, ethnicity, educational level, assessment center, Townsend deprivation index, aspirin use, non-aspirin non-steroidal anti-inflammatory drug use, vitamin supplementation, and mineral and other dietary supplementation. [b]Unhealthy lifestyle score was generated based on: 1) current smoking (score of 1); 2) excessive alcohol consumption (score of 1: >14 units/week); 3) unhealthy diet (score of 1: healthy diet score <5); 4) insufficient physical activity (score of 1: not meeting the criterium of ≥150 min of moderate activity per week, ≥75 min of vigorous activity per week, an equivalent combination, moderate activity on ≥5 d per week, or vigorous activity at least once per week); and 5) suboptimal sleep duration (score of 1: <7 or >8 h per day), ranging from 0–5 and with a higher score indicating an unhealthier lifestyle. The study participants were then categorized into three groups based on tertiles of the score: favorable (score <2), intermediate (score =2), and unfavorable (score >2) lifestyle. BMI. Body mass index; CI. Confidence interval; *HR*. Hazard ratio

Table S15 Sepsis incidence and 28-day mortality following sepsis by metabolic syndrome (MetS) component combinations in the UK Biobank[a]

MetS components	Phenotype (Combination of MetS components)	No. of participants	Sepsis incidence [*HR* (95% CI)]	28-day mortality following sepsis [*HR* (95% CI)]
Components<3	Non-MetS	242,571	Reference	Reference
Components=3	Elevated waist circumference+Hypertriglyceridemia+Elevated blood pressure	19,199	1.29 (1.19–1.40)	1.10 (0.90–1.35)
	Hypertriglyceridemia+Elevated blood pressure+Reduced HDL-cholesterol	14,901	1.04 (0.95–1.14)	1.04 (0.84–1.28)
	Elevated waist circumference+Elevated blood pressure+Reduced HDL-cholesterol	9901	1.48 (1.34–1.64)	1.60 (1.29–1.98)
	Elevated blood pressure+Hyperglycemia+Reduced HDL-cholesterol	5593	1.52 (1.36–1.71)	1.62 (1.28–2.05)
	Hypertriglyceridemia+Elevated blood pressure+Hyperglycemia	4963	1.07 (0.92–1.24)	1.04 (0.74–1.47)
	Elevated waist circumference+Elevated blood pressure+Hyperglycemia	4384	1.51 (1.31–1.75)	1.73 (1.27–2.35)
	Elevated waist circumference+Hypertriglyceridemia+Reduced HDL-cholesterol	3740	1.67 (1.40–1.99)	1.83 (1.23–2.72)
	Hypertriglyceridemia+Hyperglycemia+Reduced HDL-cholesterol	1054	1.53 (1.15–2.02)	1.14 (0.57–2.30)
	Elevated waist circumference+Hypertriglyceridemia+Hyperglycemia	822	1.44 (1.01–2.05)	1.00 (0.38–2.68)
	Elevated waist circumference+Hyperglycemia+Reduced HDL-cholesterol	778	1.70 (1.22–2.38)	1.65 (0.78–3.48)
Components=4	All components without hyperglycemia	17,463	1.55 (1.44–1.67)	1.46 (1.22–1.74)
	All components without hypertriglyceridemia	6993	2.17 (1.98–2.39)	1.91 (1.55–2.36)
	All components without reduced HDL-cholesterol	5953	1.65 (1.47–1.86)	2.10 (1.67–2.65)
	All components without elevated waist circumference	5640	1.48 (1.32–1.66)	1.50 (1.18–1.90)
	All components without elevated blood pressure	1471	1.66 (1.31–2.10)	1.17 (0.62–2.18)
Components=5	All five components	14,207	2.26 (2.11–2.43)	1.88 (1.60–2.22)

[a]Multivariable model was adjusted for age, sex, ethnicity, educational level, assessment center, Townsend deprivation index, smoking status, alcohol consumption, healthy diet, regular physical activity, sleep duration, aspirin use, non-aspirin non-steroidal anti-inflammatory drug use, vitamin supplementation, and mineral and other dietary supplementation. CI. Confidence interval; HDL. High-density lipoprotein; *HR*. Hazard ratio
